# Supplementary figures and images for: Physical interactions between specifically regulated subpopulations of the MCM and RNR complexes prevent genetic instability
Source: PLoS Genet. 2024 May 22;20(5):e1011148. doi: 10.1371/journal.pgen.1011148 (PMC11149843; doi:10.1371/journal.pgen.1011148)

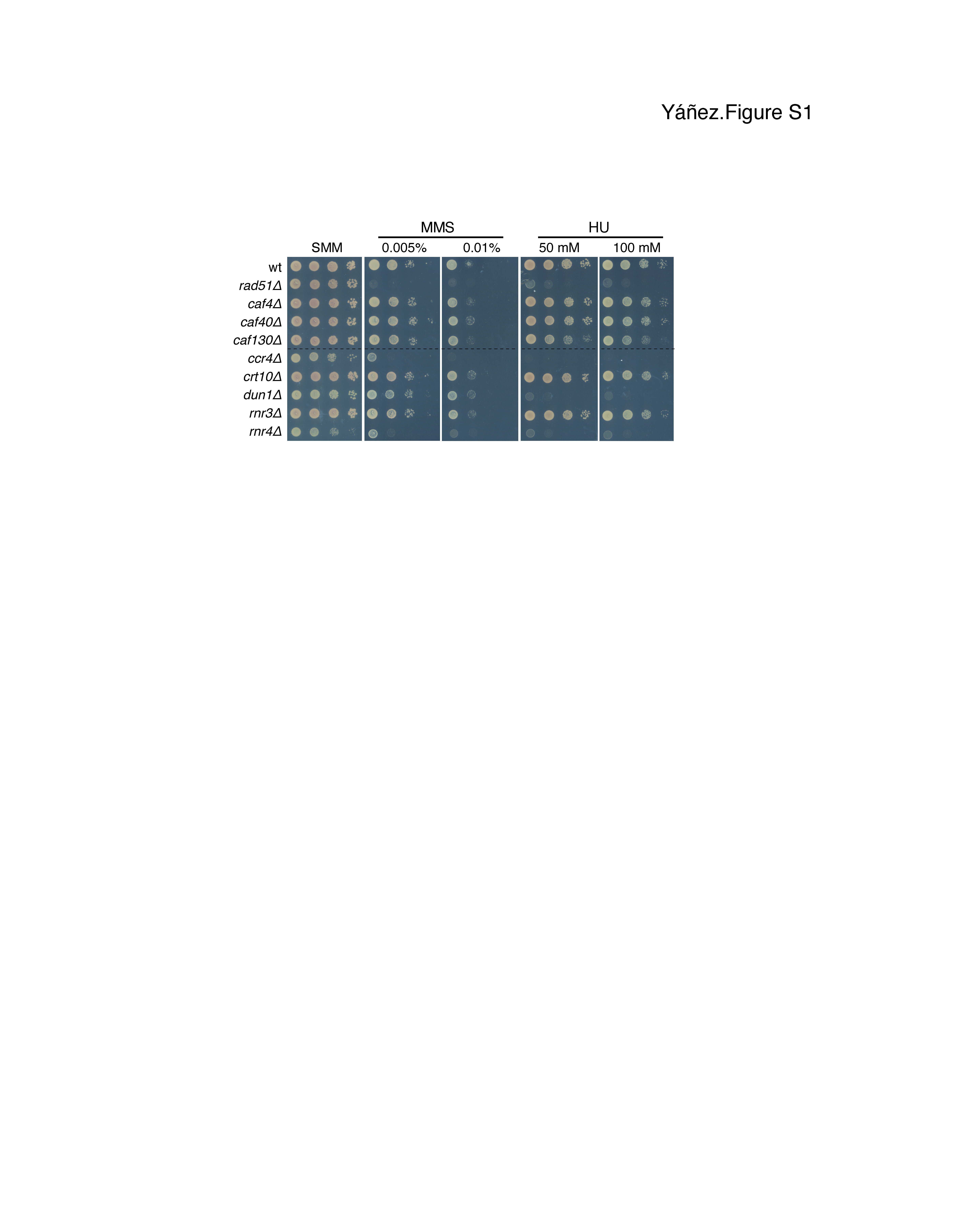

Supplement: S1 Fig — The absence of Ccr4 and Rnr4 causes MMS and HU sensitivity as determined by ten-fold serial dilutions at the indicated concentrations. The experiment was repeated twice with similar results. (TIFF) [file pgen.1011148.s001.tiff]

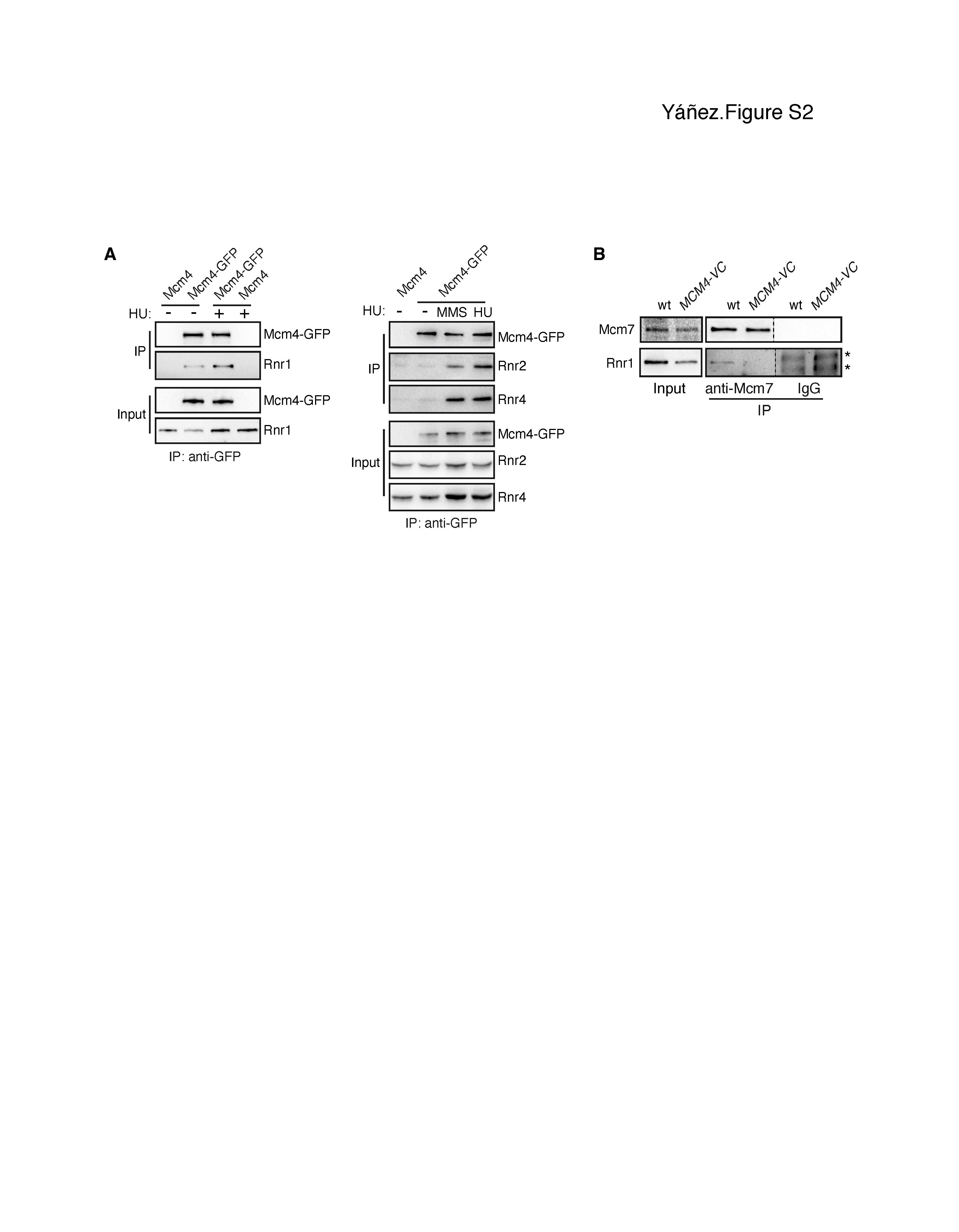

Supplement: S2 Fig — (A) HU promotes MCM/RNR interactions, as determined by CoIP and western blot in asynchronous cultures treated with 0.2M HU for 2 hours. Untreated and treated cells with 0.025% MMS for 2 hours were included as control. (B) Mcm7 interacts with Rnr1, as determined by CoIP and western blot in asynchronous cultures of wild-type cells. This interaction is lost in the MCM-VC mutant. Asterisks represent unspecific bands. (TIFF) [file pgen.1011148.s002.tiff]

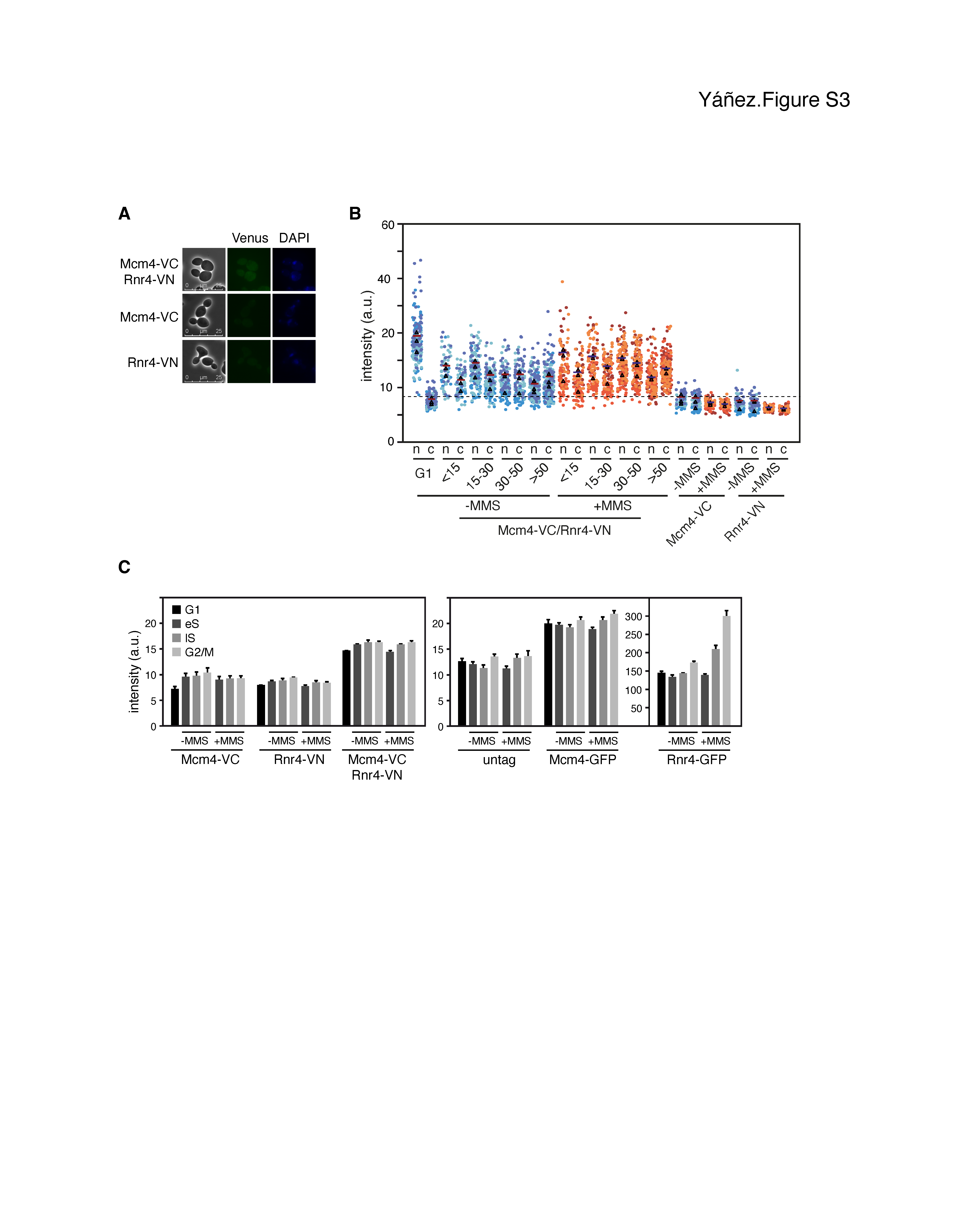

Supplement: S3 Fig — (A-C) BiFC analysis of the subcellular location and intensity of the Mcm4VC/Rnr4-VN interaction during the cell cycle in cells synchronized in G1 and released into fresh medium in the absence and presence of 0.025% MMS. (A) Representative images of dividing cells expressing Mcm4-VC and/or Rnr4-VN. (B) Intensity of the Mcm4VC/Rnr4-VN fluorescence signal in the nucleus and cytoplasm of cells grouped according to the bud-to-mother size ratio to compare similar stages of DNA replication with and without DNA damage. The Venus signal was calculated in equivalent areas of the nucleus and the cytoplasm. The SuperPlot shows the mean and SEM from three independent experiment (represented by color dots). (C) Intensity of the Mcm4-VC/Rnr4-VN fluorescence signal as determined by cell sorting analysis. Cells were grouped in G1, early and late S phase (eS and lS) and G2/M according to the presence and size of the bud. The amount of the MCM and Rnr2/Rnr4 complexes was followed by quantifying the GFP signal in cells expressing Mcm4-GFP and Rnr4-GFP under the same experimental conditions. The mean and SEM of 4–7 fluctuations tests are shown. (TIFF) [file pgen.1011148.s003.tiff]

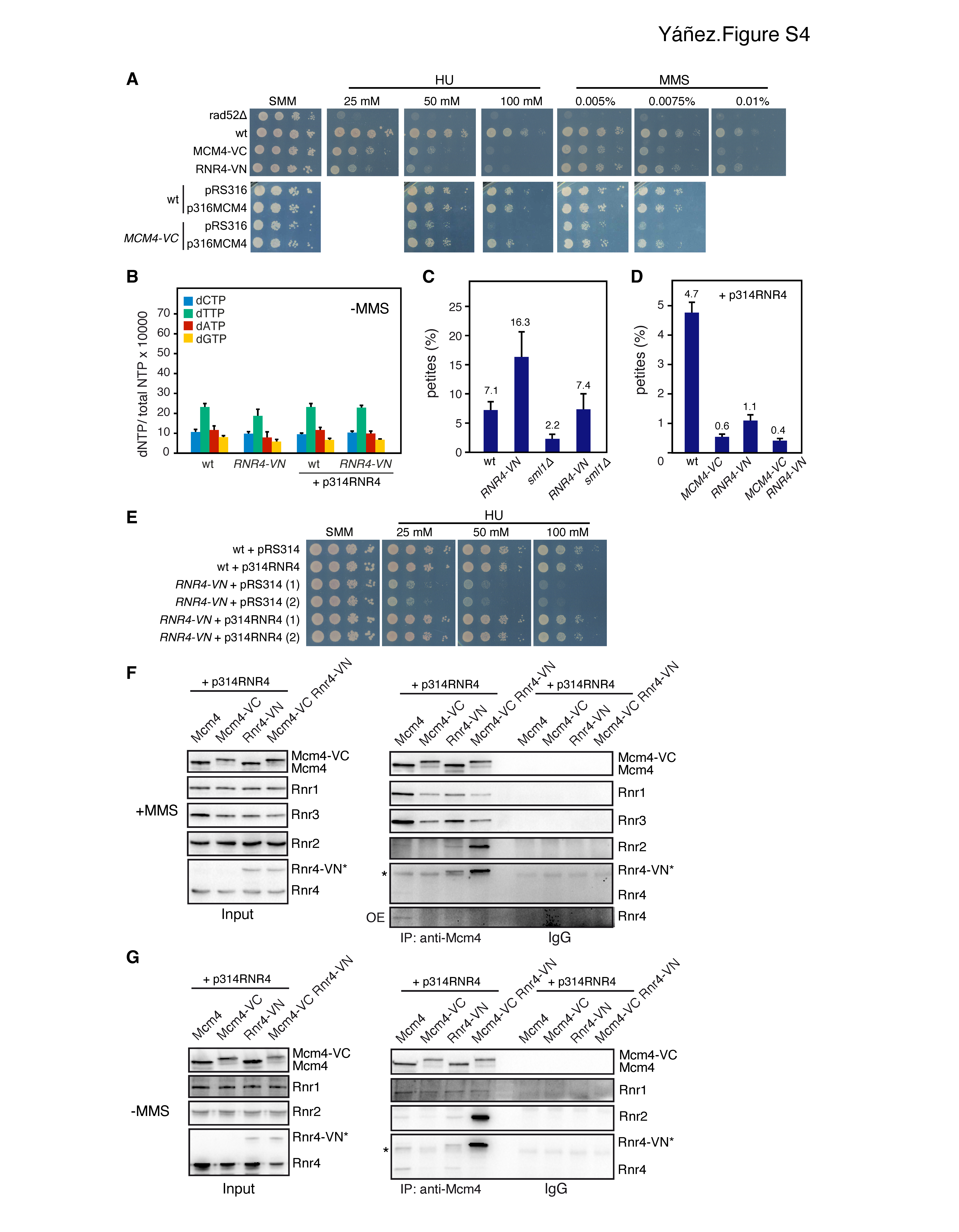

Supplement: S4 Fig — (A) The expression of Mcm4-VC or Rnr4-VN causes HU and MMS sensitivity as determined by ten-fold serial dilutions at the indicated concentrations (top panels). The MCM4-VC allele is recessive for HU and MMS sensitivity, as determined in cells transformed with a centromeric plasmid expressing Mcm4 from its own promoter (p316MCM4) or an empty vector (pRS316) (bottom panels). The experiments were repeated twice with similar results. (B) Levels of dNTPs in RNR4-VN and wild-type cells transformed or not with plasmid p314RNR4. The mean and SEM of four independent experiments are shown. (C-D) Frequency of petite formation in the indicated strains. The mean and SEM of 3 fluctuations tests are shown. (E) The HU sensitivity of the RNR4-VN mutant can be complemented with a Rnr4 expressing plasmid as determined by ten-fold serial dilutions at the indicated concentrations. The experiments were repeated twice with similar results. (F-G) Analysis of the MCM/RNR interactions in cells expressing Mcm4-VC and/or Rnr4-VN transformed with plasmid p314RNR4. Effect of tagging Mcm4 and Rnr4 with VC and VN, respectively, on the physical interactions between the MCM and RNR complexes in cells transformed with plasmid p314RNR4, as determined by CoIP and western blot in asynchronous cultures treated (F) or not (G) with 0.025% MMS for 2 hours. Asterisks indicate unspecific bands. Rnr4-VN* indicates a slow-migrating Rnr4-VN form. An over-exposition (OE) of the Rnr4 IP signals is shown in the bottom panel. CoIP analyses were performed twice with similar results. (TIFF) [file pgen.1011148.s004.tiff]

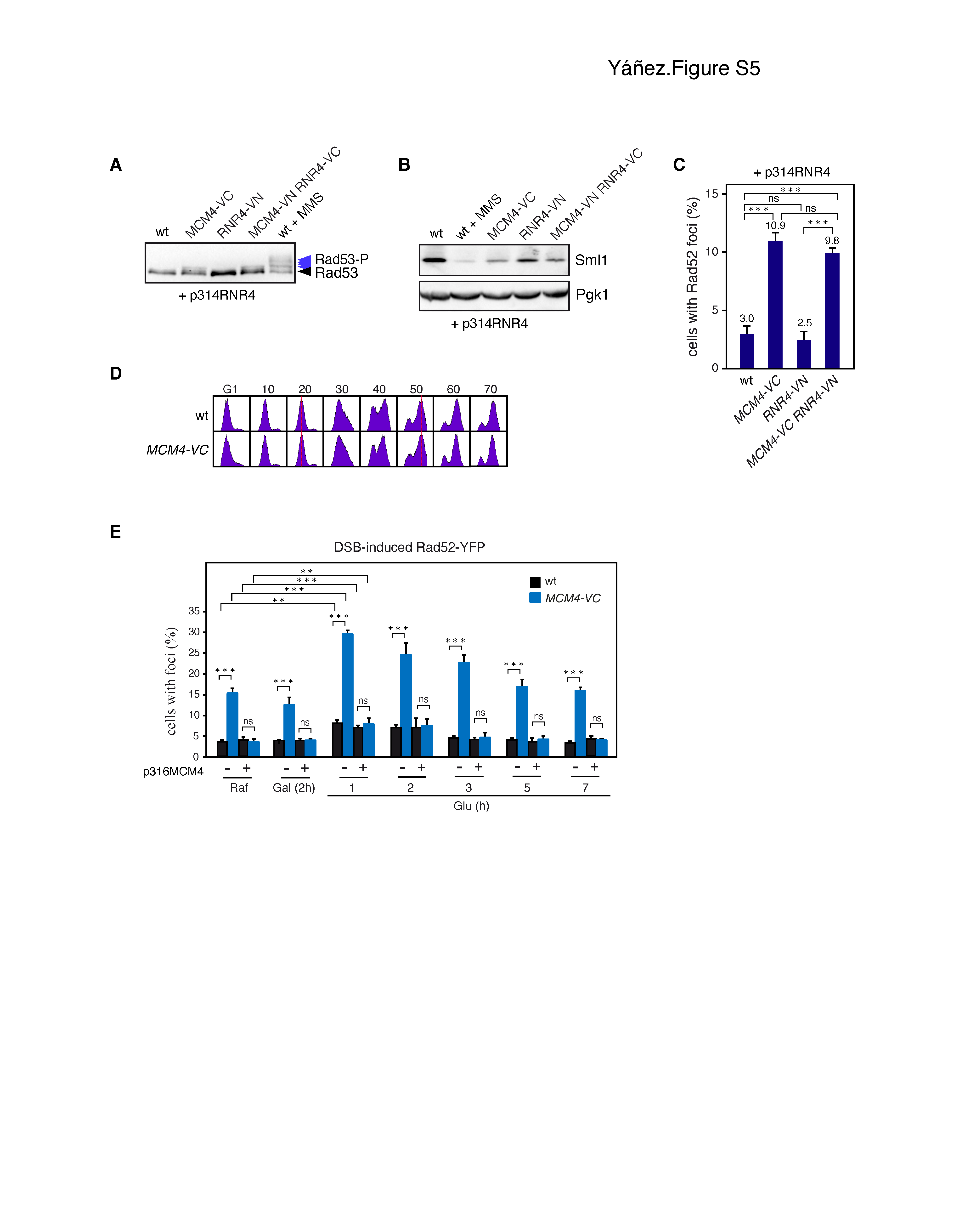

Supplement: S5 Fig — (A-B) Rad53 phosphorylation (A) and Sml1 levels (B) at asynchronous cultures of the indicated strains transformed with plasmid p314RNR4 in the absence of genotoxic agents. A wild-type culture incubated with 0.025% MMS for 1 hour was included as positive control. (C) Spontaneous accumulation of Rad52 foci at asynchronous cultures of the indicated strains transformed with plasmid p314RNR4. (D) Cell cycle progression of MCM-VC and wild-type strains synchronized in G1 and released into S phase, as determined by cell sorting analysis. (E) The MCM4-VC allele is recessive for the accumulation of spontaneous and HO-induced Rad52 foci, as determined in cells transformed with a centromeric plasmid expressing Mcm4 from its own promoter (p316MCM4) or an empty vector (pRS316). The kinetics of HO-induced Rad52 foci formation and resolution was performed as indicated in Fig 5B. (C, E) The mean and SEM of three independent experiments are shown. Asterisks indicate statistically significant differences according to an unpaired two-tailed Student’s t-test (three asterisks represent a P-values <0.001). (TIFF) [file pgen.1011148.s005.tiff]

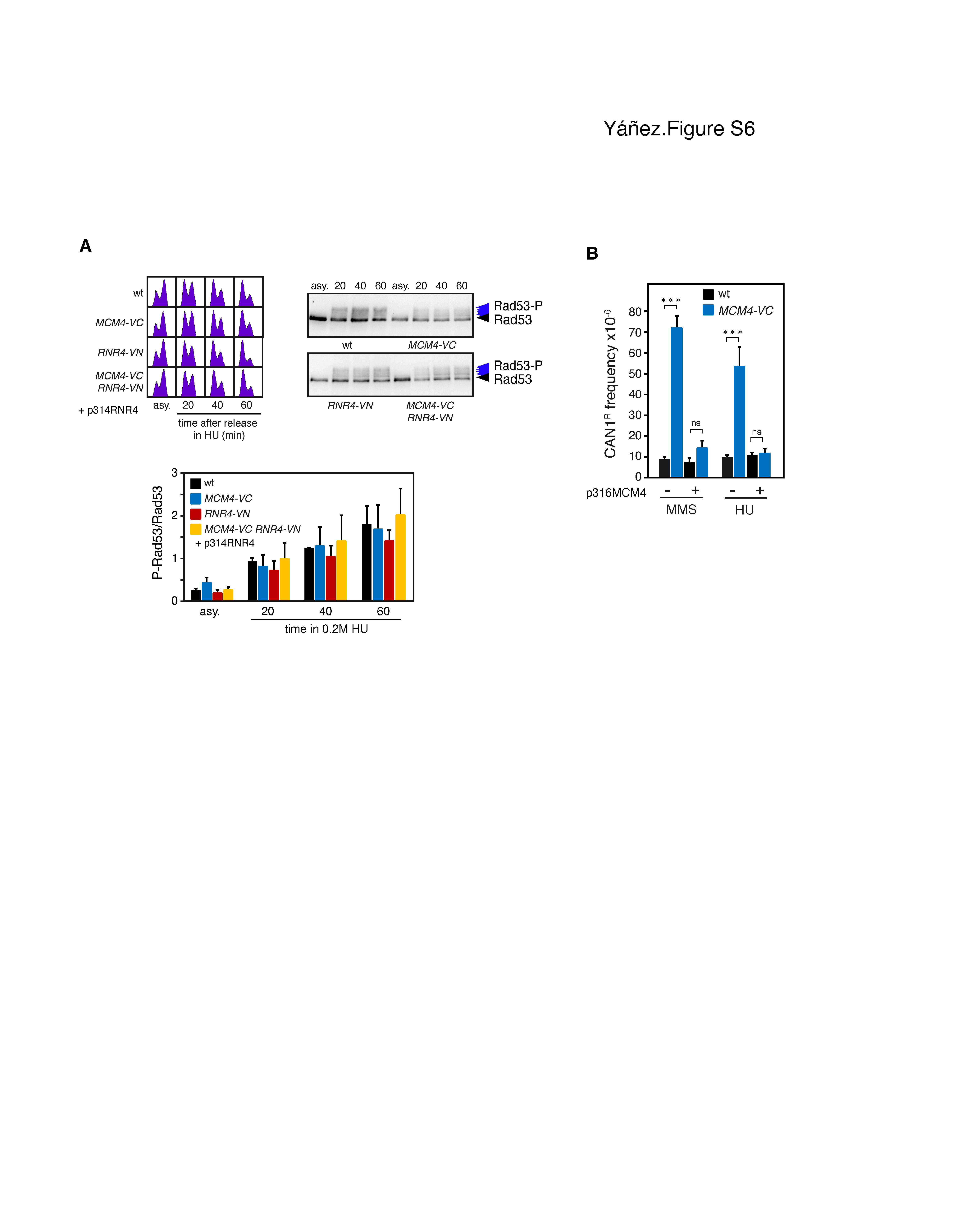

Supplement: S6 Fig — (A) Checkpoint activation is proficient in MCM4-VC mutants. Checkpoint activation in MCM4-VC and RNR4-VN mutants, as determined by western blot against Rad53 in asynchronous cultures treated with 0.2M HU for 60 minutes. Cell cycle progression was determined by cell sorting analysis. The P-Rad53/Rad53 ratio was determined by dividing the upper bands signal (Rad53-P) by the lower band signal (Rad53). Experiments were performed with cells transformed with plasmid p314RNR4. (B) The MCM4-VC allele is recessive for hypermutagenesis, as determined in MCM4-VC and wild-type cells transformed with a centromeric plasmid expressing Mcm4 from its own promoter (p316MCM4) or an empty vector (pRS316). The frequency of mutagenesis was determined from colonies grown with 0.0075% MMS or 50mM HU. The mean and SEM of three independent experiments are shown. Asterisks indicate statistically significant differences according to an unpaired two-tailed Student’s t-test (three asterisks represent a P-values <0.001). (TIFF) [file pgen.1011148.s006.tiff]

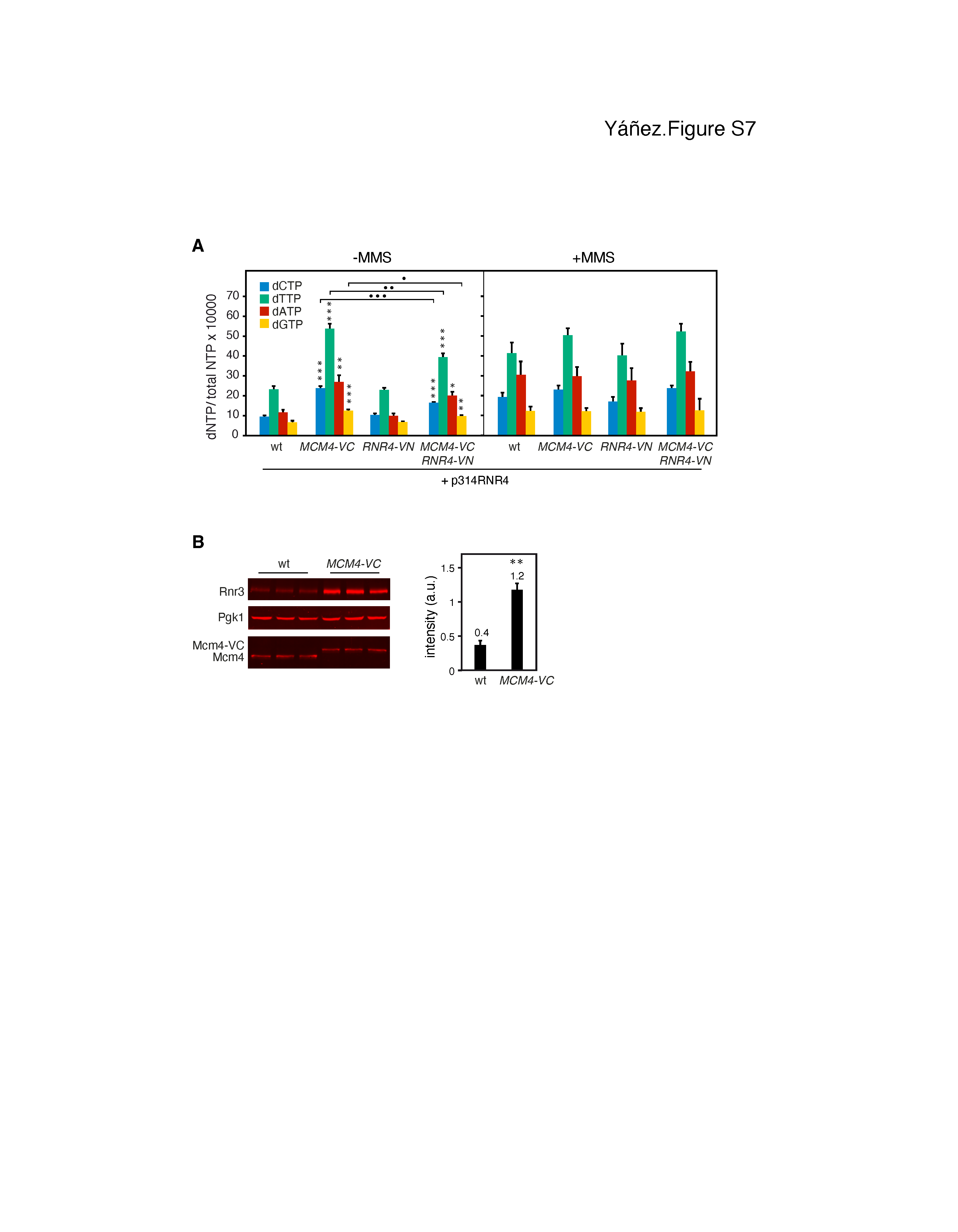

Supplement: S7 Fig — (A) Levels of dNTPs in MCM4-VC and RNR4-VN mutants in the absence and presence of 0.05% MMS, as determined in mid-log phase asynchronous cultures of the indicated strains transformed with plasmid p314RNR4. The mean and SEM of four independent experiments are shown. Asterisks (relative to the wild type) and dots (between the indicated values) show statistically significant differences according to an unpaired two-tailed Student’s t-test (one, two and three asterisks represent P-values <0.05, <0.01 and <0.001, respectively). (B) The amount of Rnr3 is increased in MCM4-VC cells, as determined by western blot analysis from asynchronous cultures of MCM4-VC and wild-type cells. The amount of Rnr3 was normalized to the amount of Pgk1. The mean and SEM of three independent experiments are shown. Asterisks indicate statistically significant differences according to an unpaired two-tailed Student’s t-test (two asterisks represent a P-values <0.01). (TIFF) [file pgen.1011148.s007.tiff]

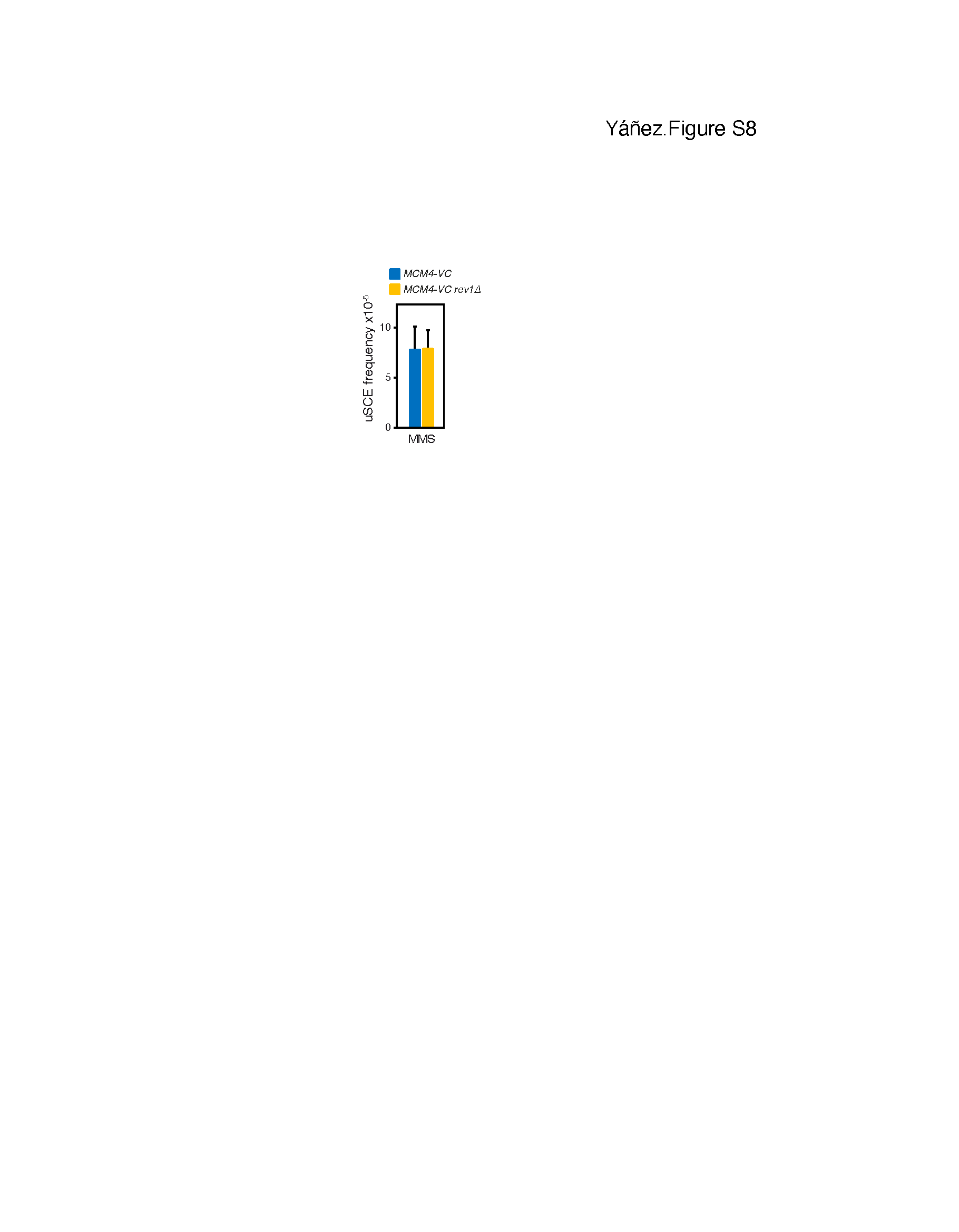

Supplement: S8 Fig — The frequency of HR was determined from colonies grown in the presence of 0.0075% MMS. The mean and SEM of 4 fluctuations tests are shown. (TIFF) [file pgen.1011148.s008.tiff]

B

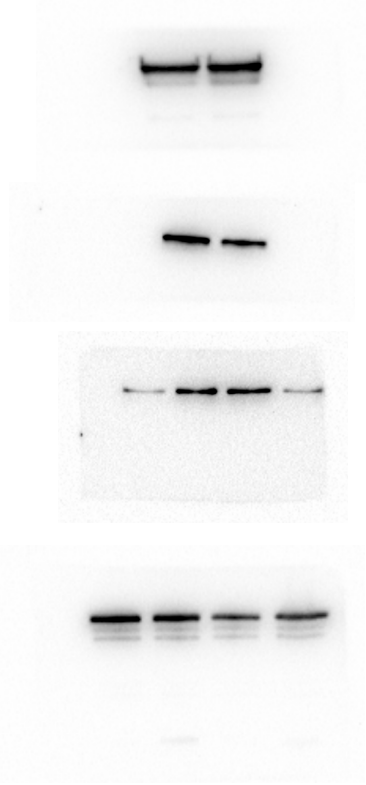

C

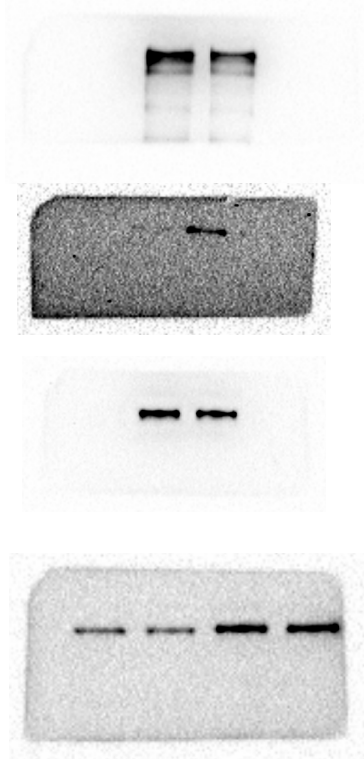

D

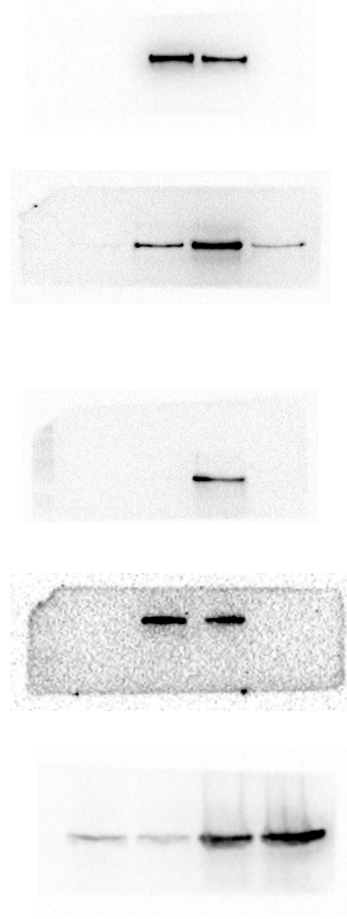

E

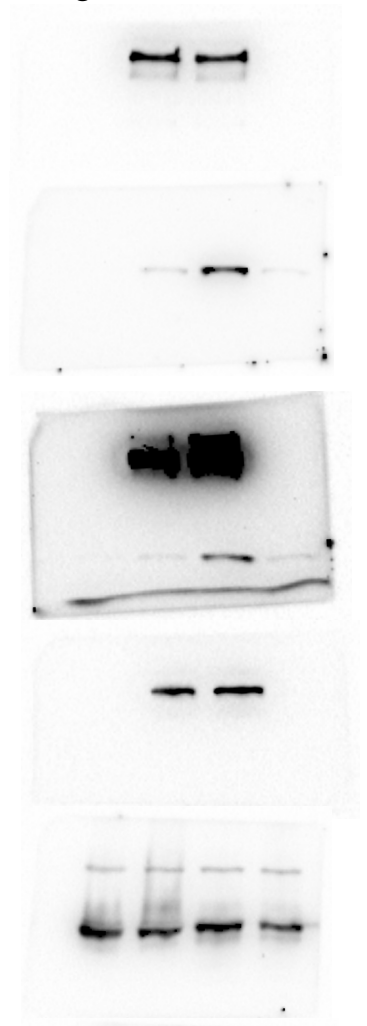

F

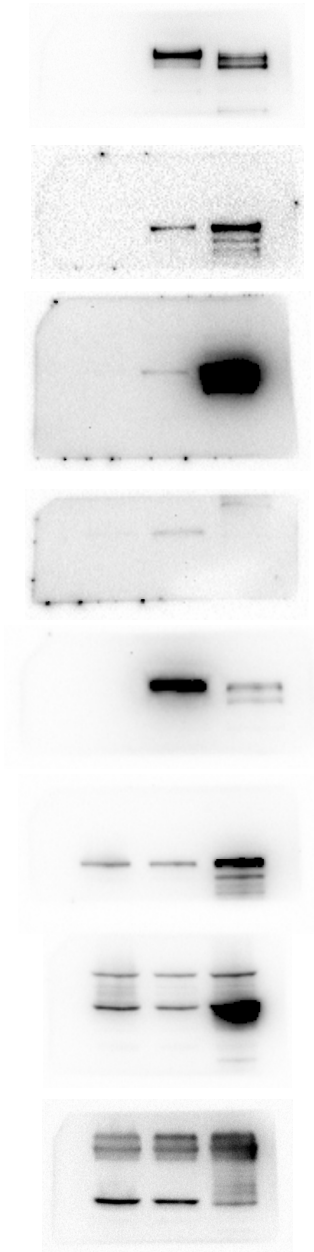

G

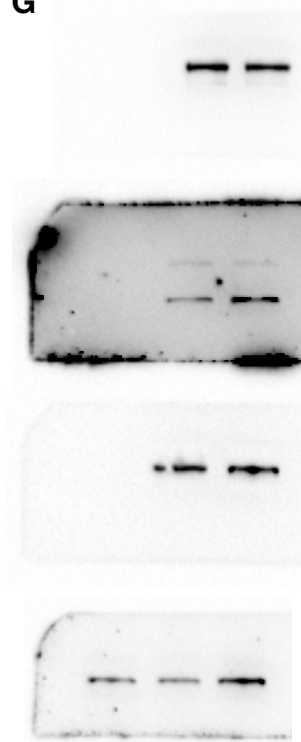

H

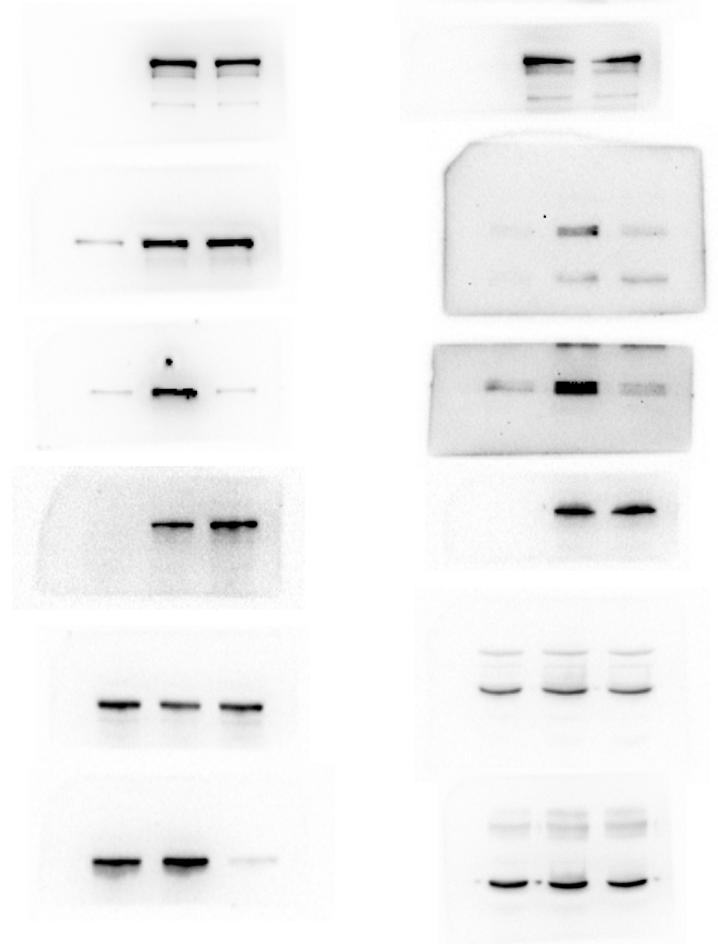

D

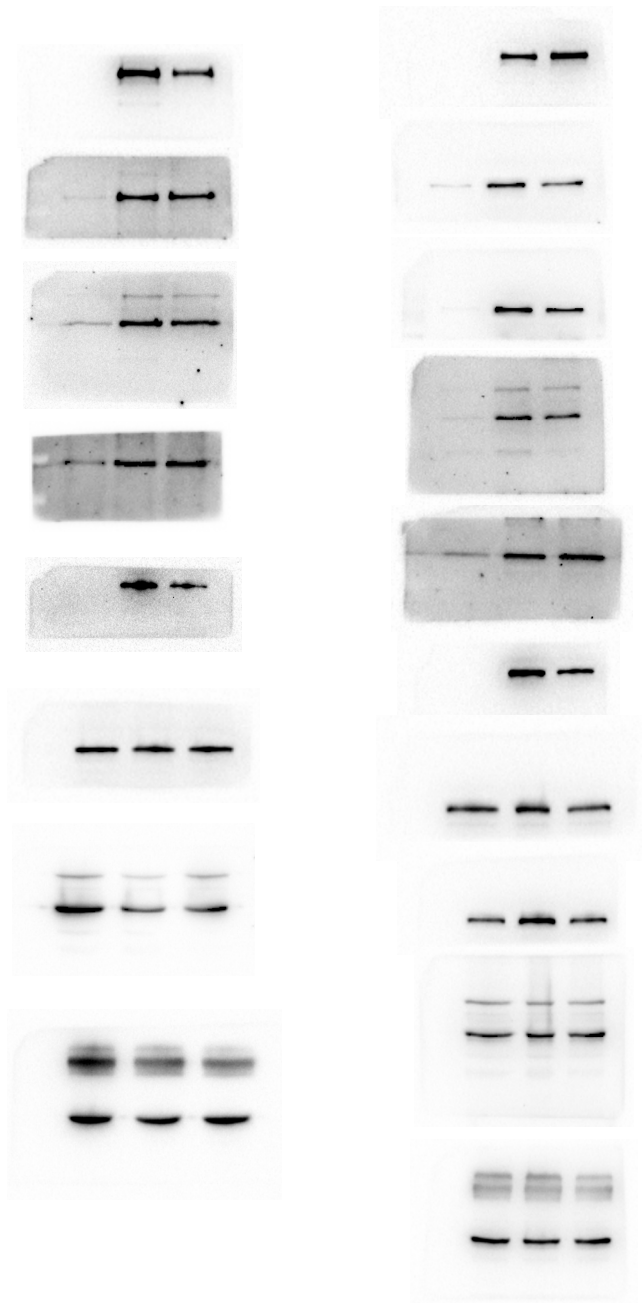

E

Yáñez.Raw data Fig2D-F

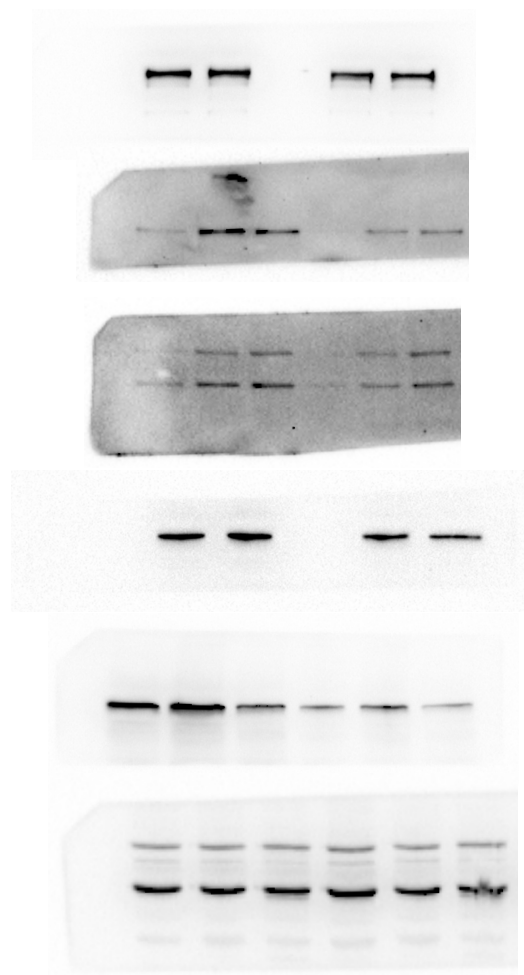

F

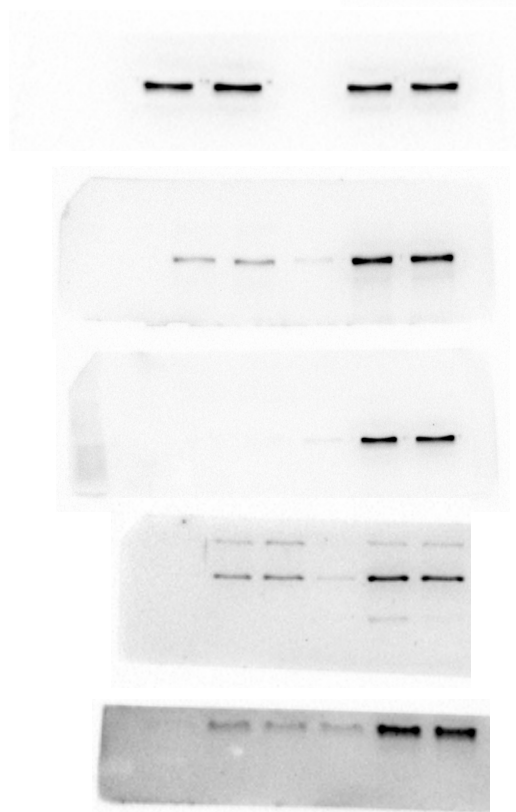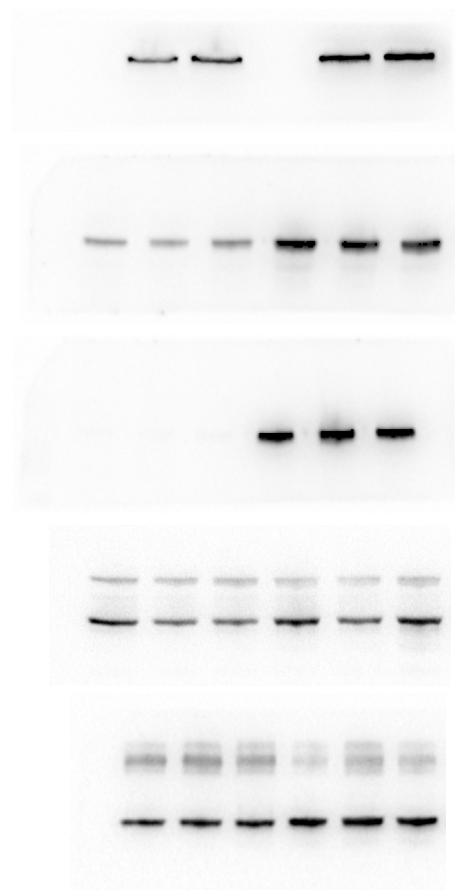

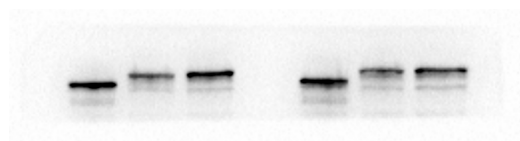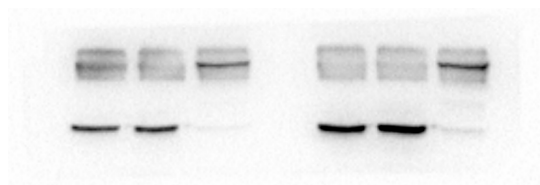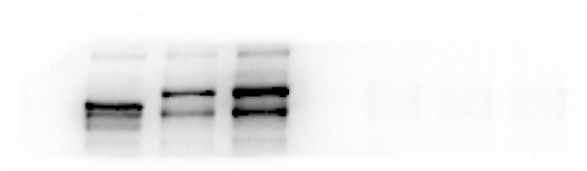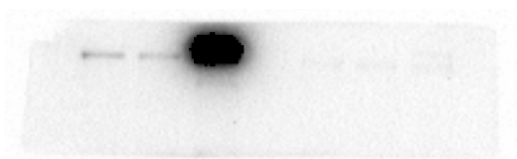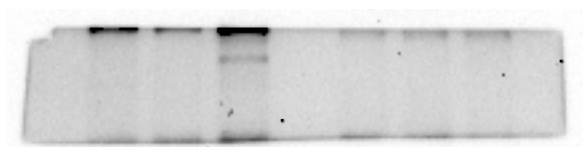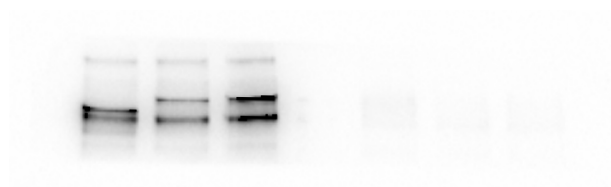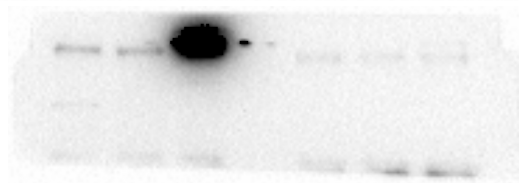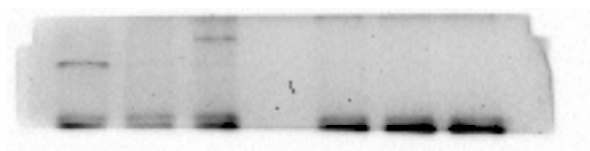

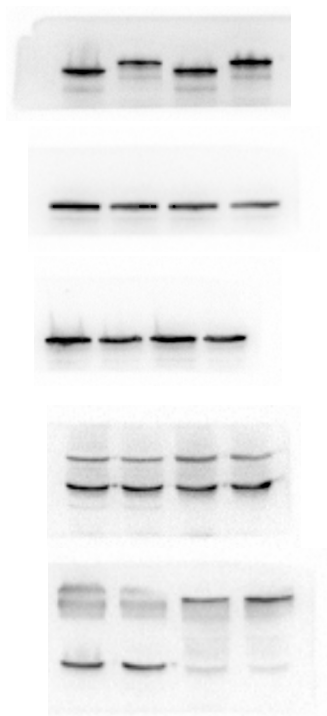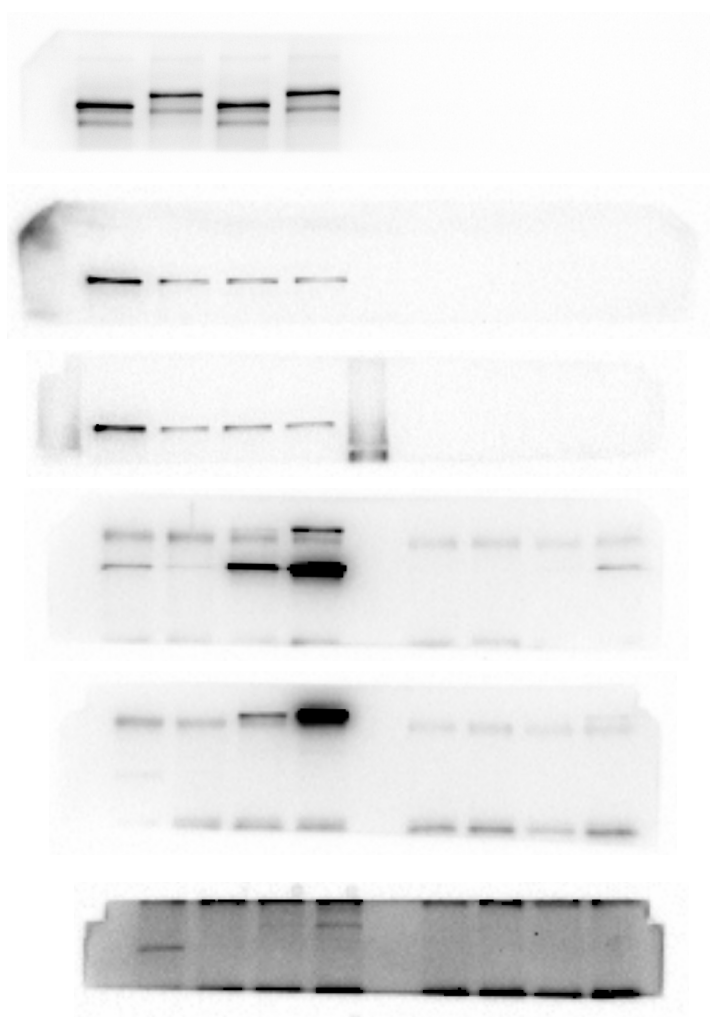

D

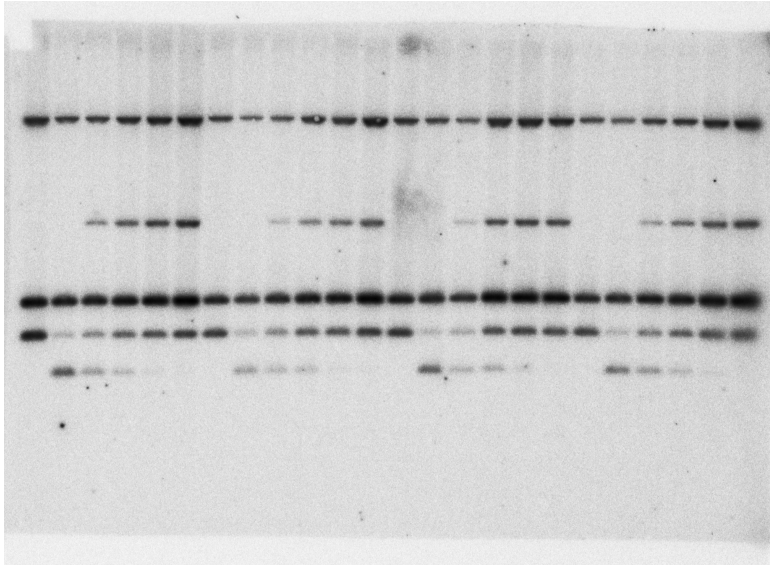

F

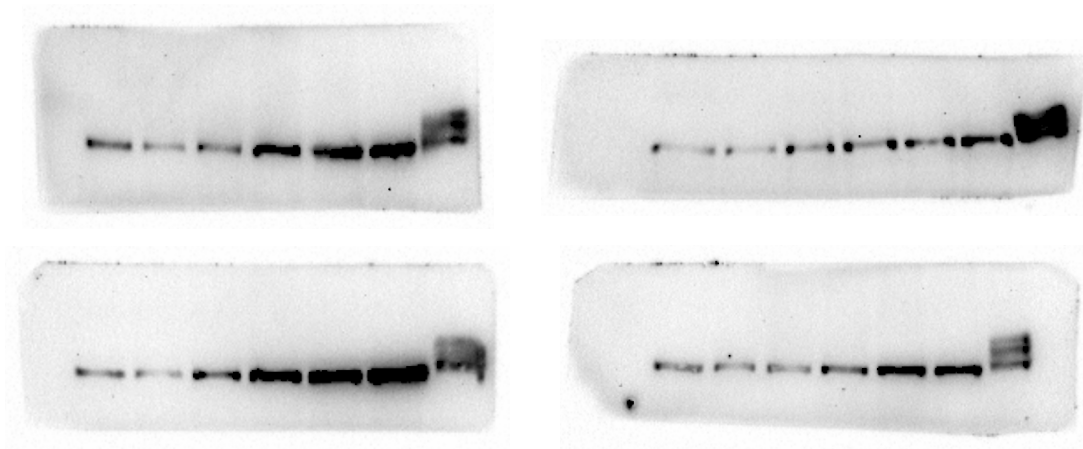

A

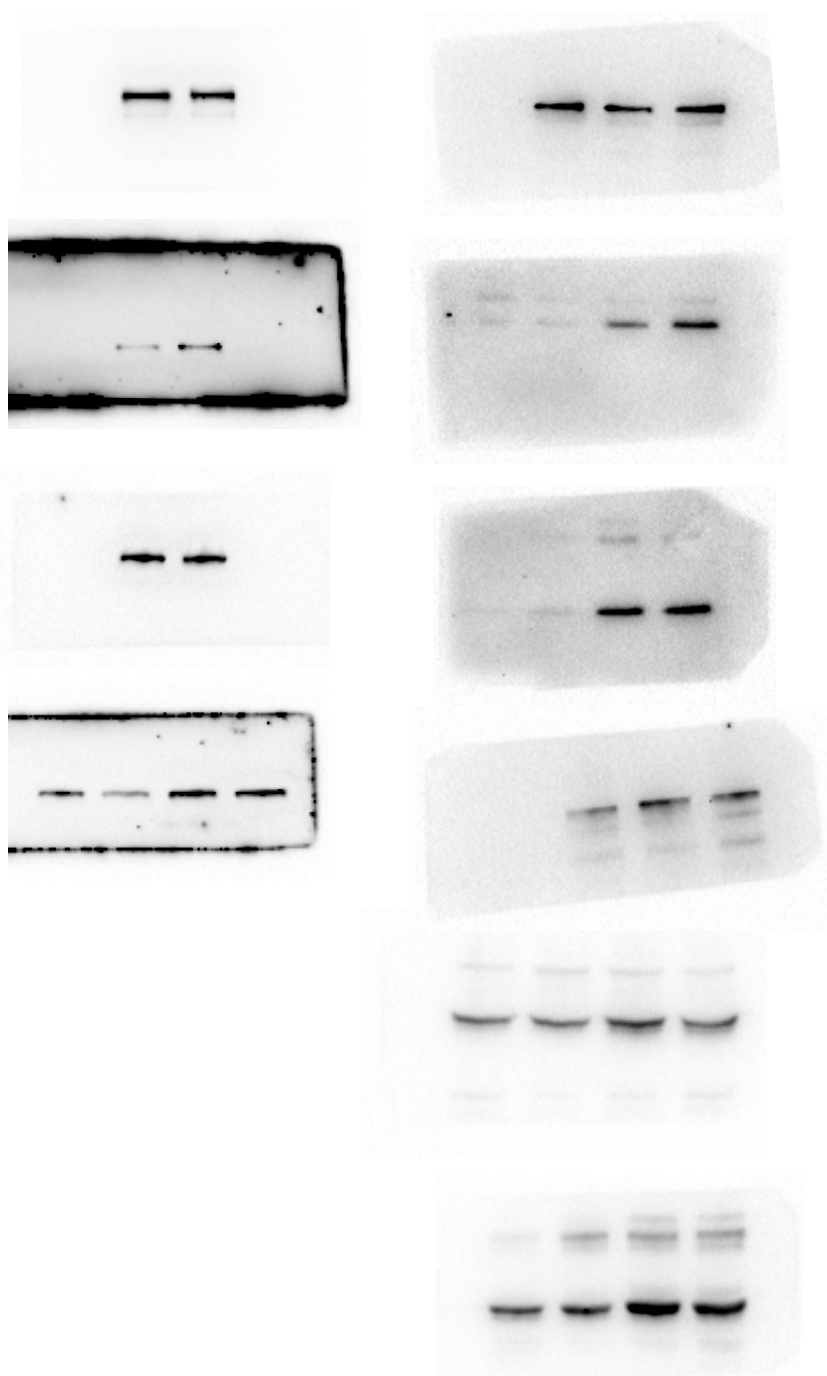

B

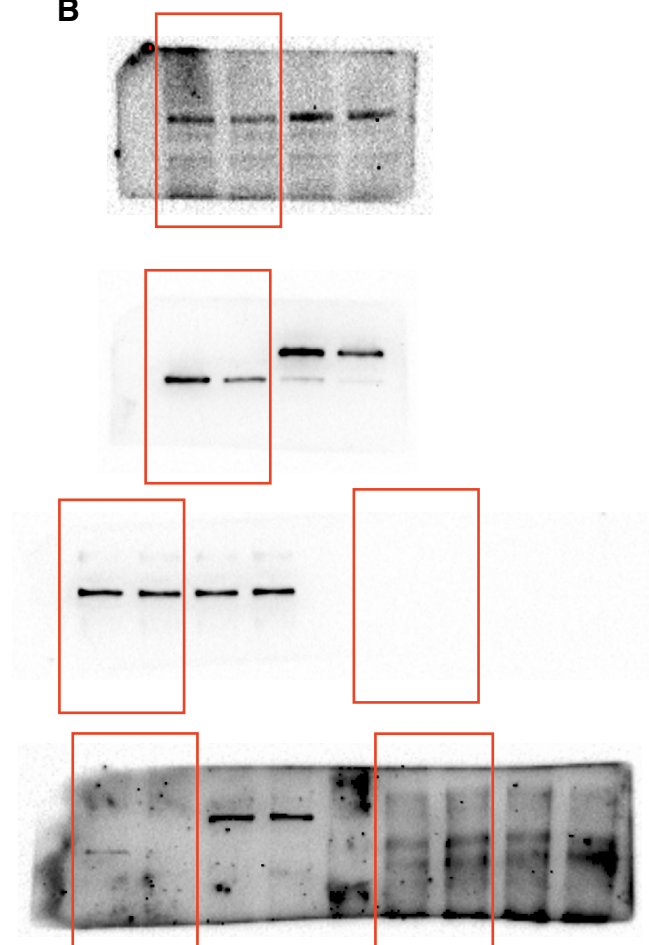

F

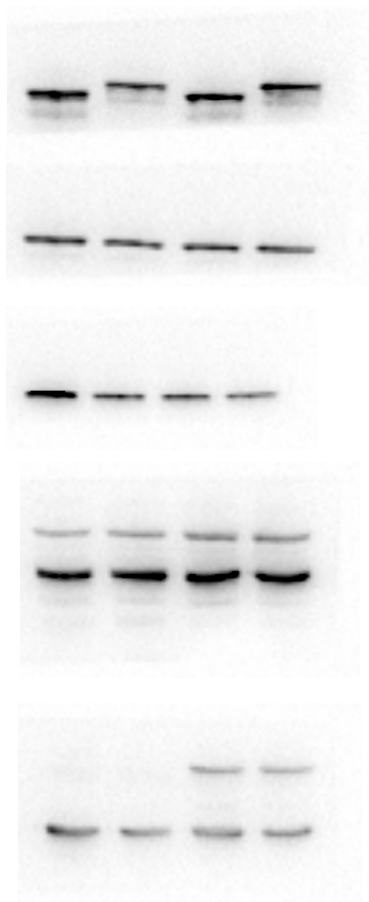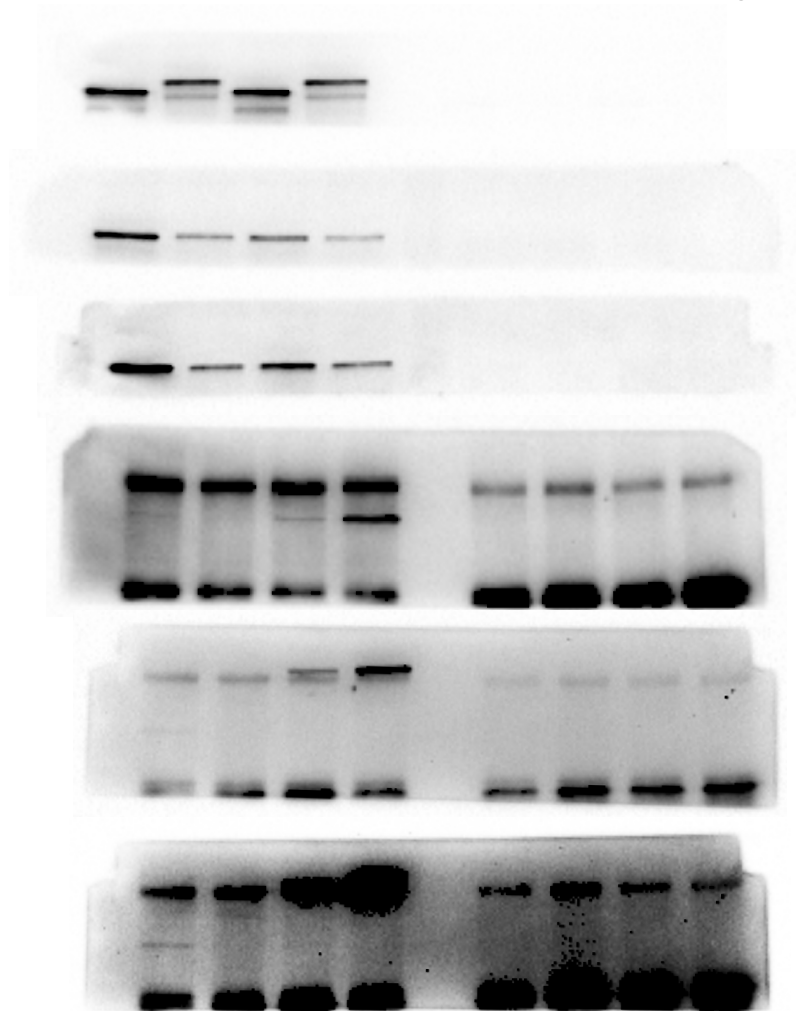

G

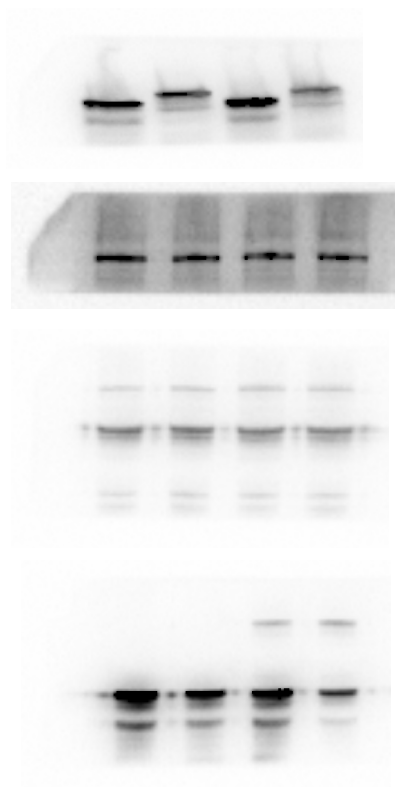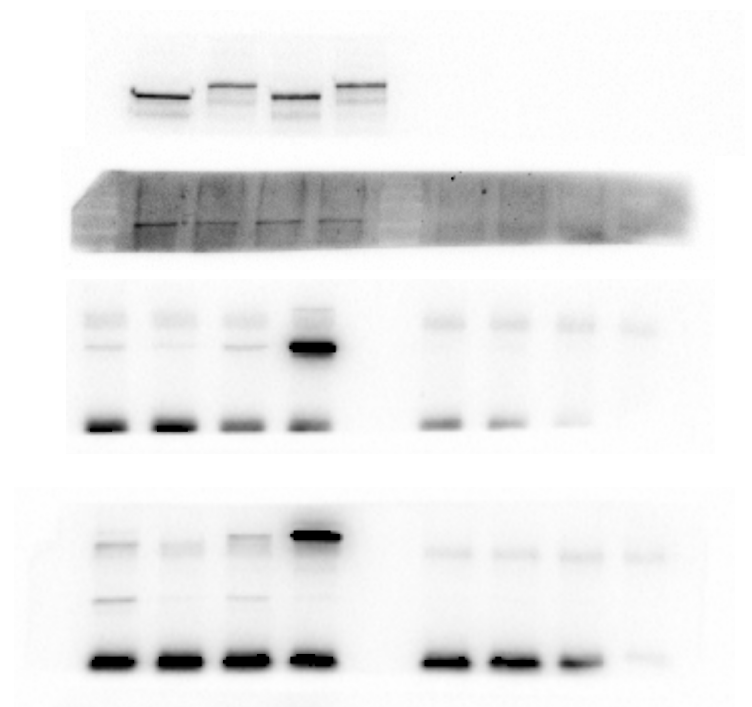

S5A

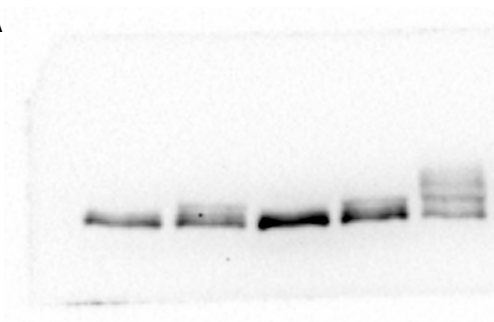

S5B

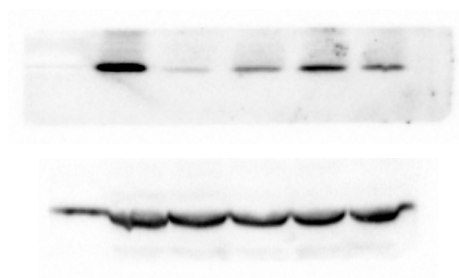

S6A

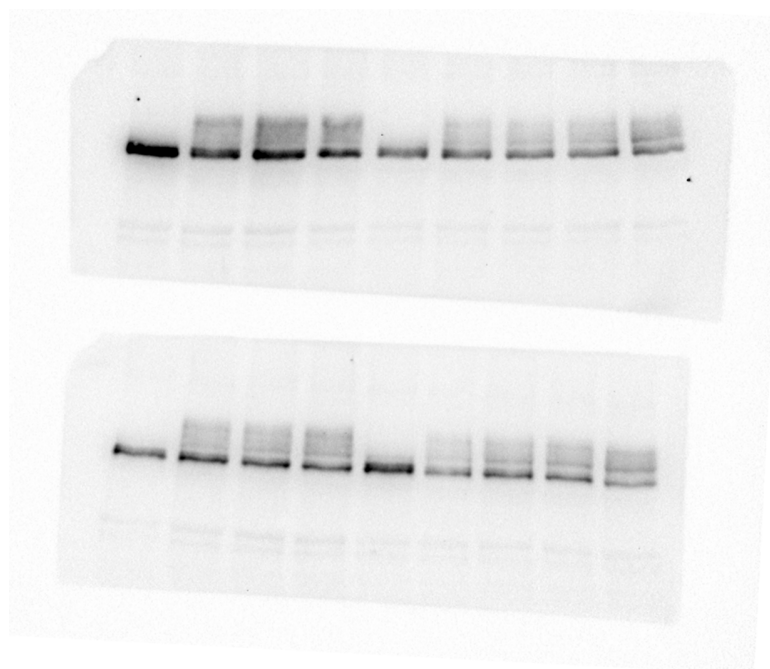

S7B

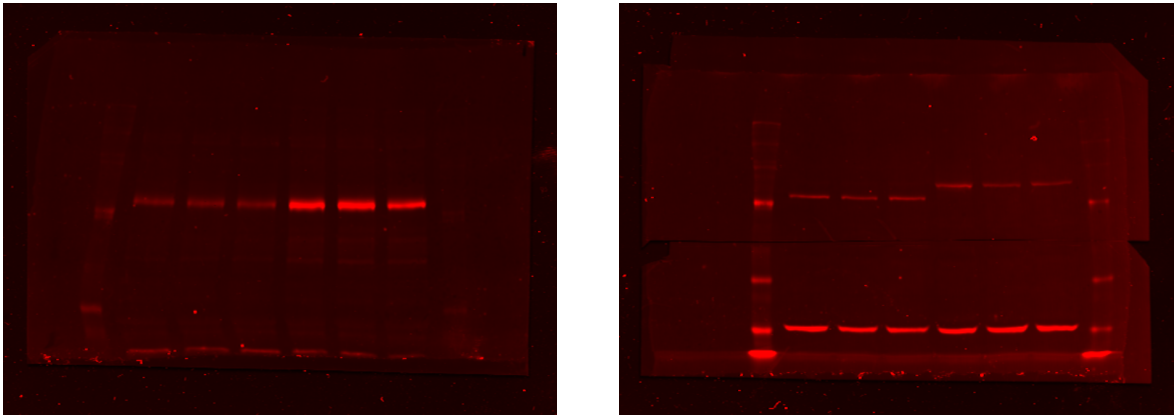

Supplement: S9 Fig — Original blots for the indicated figure panels are shown. (PDF) [file pgen.1011148.s009.pdf]
