# Supplementary material for: Physical interactions between specifically regulated subpopulations of the MCM and RNR complexes prevent genetic instability
Source: PLoS Genet. 2024 May 22;20(5):e1011148. doi: 10.1371/journal.pgen.1011148 (PMC11149843; doi:10.1371/journal.pgen.1011148)
Supplement: S1 Table — Strains, genotypes, references and Figures panels where they have been used are indicated. (DOCX) [file pgen.1011148.s010.docx]

| **Strain**  **Table S1. *Saccharomyces cerevisiae* strains used in this study** | ***Genotype*** | **Ref.** | **Figure** |
| --- | --- | --- | --- |
| W303-1a | *MATa leu2-3,112 trp1-1 ura3-1 ade2-1 his3-11,15 can1-100 BAR1 RAD5* | [1] | 1A, 3B, 4, 5B, 5D, 5E, 5F, 6A, 6B, 6C, S1, S4B, S4C, S4D, S4F, S4G, S5C, S5B, S5E, S6A, S7A, S7B |
| wMCM4GFPpep4-7A | *MATa MCM4GFP::URA3 pep4∆::ADE2* | [2] | 1A, 1D, 1E, 1F, 1H, 2E, 2F, 3A, S2A, S3C |
| wCcr4HA-5D | *MATa CCR4HA::HIS3 pep4∆::ADE2* | This work | 1B |
| wCcr4HAMcm4GFP-1B | *MATa CCR4HA::HIS3 MCM4GFP::URA3 pep4∆::ADE2* | This work | 1B |
| wDun1HA-4D | *MATa DUN1HA::HIS3 pep4∆::ADE2* | This work | 1C |
| wDun1HAMcm4GFP-5D | *MATa DUN1HA::HIS3 MCM4GFP::URA3 pep4∆::ADE2 rad5-535* | This work | 1C |
| w303pep4∆ | *MATa pep4∆::ADE2* | This work | 1D, 1E, 1F,1H, 2D, 2E, 2F, S2A, S3C |
| wM4Gr4∆p-12D | *MATa MCM4GFP::URA3 rnr4∆::Kan pep4∆::ADE2* | This work | 1F |
| wRnr3HA-7D | *MATalfa RNR3HA::HIS3 pep4∆::ADE2 rad5-535* | This work | 1G |
| wRnr3HAMcm4GFP-9A | *MATalfa RNR3HA::HIS3 MCM4GFP::URA3 pep4∆::ADE2 rad5-535* | This work | 1G |
| wM4GR3HAdun1-5C | *MATalfa MCM4GFP::URA3 RNR3HA::HIS3 pep4∆::ADE2 dun1∆::Kan* | This work | 1G |
| wM4Gdun1∆p-3A | *MATa MCM4GFP::URA3 dun1∆::Kan pep4∆::ADE2* | This work | 1H |
| wM4gR4c-5C | *MATa MCM4GFP::URA3 RNR4Cherry::Nat* | This work | 2A, 2B, 2C |
| wM4dwR4-1A | *MATa MCM4GFP::URA3 RNR4Cherry::Nat dif1∆::Kan wtm1∆::Kan* | This work | 2A, 2B |
| wR4M4svnls-1C | *MATa RNR4Cherry::Nat MCM4GFP::svnls3A2::Kan pep4∆::ADE2* | This work | 2A, 2C |
| wR4M4NLS-6C | *MATa RNR4Cherry::Nat MCM4GFP::NLS::Kan pep4∆::ADE2* | This work | 2A, 2C |
| wM4NLS-16A | *MATa MCM4GFP::NLS::Kan* | This work | 2D |
| wM4svnls-3B | *MATa MCM4GFP::svnls3A2::Kan* | This work | 2D |
| wM4dw-1D | *MATa MCM4GFP::URA3 dif1∆::Kan wtm1∆::Kan* | This work | 2E, 2F |
| wM4R4-1C | *MATa MCM4VC::HIS3 RNR4VN::Kan bar1∆::LEU2* | This work | 3A, S3A, S3B, S3C |
| wRnr4Cherry-3 | *MATa RNR4Cherry::Nat* | This work | 3A |
| wMcm4vc-1B | *MATa MCM4VC::HIS3 bar1∆::LEU2* | This work | 3B, 4, S3A, S3B, S3C, S4D, S4F, S4G, S5A, S5D |
| wM4R4-3D | *MATa MCM4VC::HIS3 RNR4VN::Kan* | This work | 3B, 3C, 4, 5B, 5C, 5D, 5E, 5F, 6A, 6B, 6C, S4F, S4G, S5C, S6A, S7A |
| wM4R4dw-30B | *MATa MCM4VC::HIS3 RNR4VN::Kan dif1∆::Kan wtm1∆::Kan* | This work | 3C |
| wR4-14A | *MATa RNR4VN::Kan* | This work | 4, 5B, 5D, 5E, 5F, 6A, 6B, 6C, S4B, S4F, S4G, S5C, S6A, S7A |
| wM4-3A | *MATa MCM4VC::HIS3* | This work | 5B, 5C, 5D, 5E, 5F, 6A, 6B, 6C, S5C, S5E, S6A, S7A, S7B |
| wRFA1YFP-1A | *MATa RFA1-8ala-YFP bar1∆::LEU2* | This work | 5G, 5H |
| wR1YM4VC-13D | *MATa MCM4VC::HIS3 RFA1-8ala-YFP bar1∆::LEU2* | This work | 5G, 5H |
| wR1YR4VN-1B | *MATa RNR4VN::Kan RFA1-8ala-YFP bar1∆::LEU2* | This work | 5G, 5H |
| wR1YM4VCR4VN-10B | *MATa MCM4VC::HIS3 RNR4VN::Kan RFA1-8ala-YFP bar1∆::LEU2* | This work | 5G, 5H |
| wSCE-HO-1A | *MATa-inc trp1::(his3∆3'::HOcs-his3∆5')* | This work | 6D |
| wSCE-HO-12D | *MATa-inc trp1::(his3∆3'::HOcs-his3∆5') bar1∆::LEU2* | This work | 6D |
| wSCE-HO-M4VC-8D | *MATa-inc trp1::(his3∆3'::HOcs-his3∆5') MCM4VC::Hyg bar1∆::LEU2* | This work | 6D |
| wSCE-HO-M4VC-10C | *MATa-inc trp1::(his3∆3'::HOcs-his3∆5') MCM4VC::Hyg bar1∆::LEU2* | This work | 6D |
| wSCE-HO-R4VN-7C | *MATa-inc trp1::(his3∆3'::HOcs-his3∆5') RNR4VN::Kan* | This work | 6D |
| wSCE-HO-R4VN-11B | *MATa-inc trp1::(his3∆3'::HOcs-his3∆5') RNR4VN::Kan bar1∆::LEU2* | This work | 6D |
| wSCE-HO-M4R4-2D | *MATa-inc trp1::(his3∆3'::HOcs-his3∆5') MCM4VC::Hyg RNR4VN::Kan* | This work | 6D |
| wSCE-HO-M4R4-8A | *MATa-inc trp1::(his3∆3'::HOcs-his3∆5') MCM4VC::Hyg RNR4VN::Kan* | This work | 6D |
| w303CAN-7C | *MATa bar1∆::LEU2 CAN* | This work | 6E, 6F, S4A, S6B |
| wM4CAN-5A | *MATa MCM4VC::HIS3 bar1∆::LEU2 CAN* | This work | 6E, 6F,  S4A, S6B |
| wR4CAN-1D | *MATa RNR4VN::Kan bar1∆::LEU2 CAN* | This work | 6E |
| wR4M4CAN-2D | *MATa RNR4VN::Kan MCM4VC::HIS3 bar1∆::LEU2 CAN* | This work | 6E |
| wrev1CAN-6C | *MATa rev1∆::Nat bar1∆::LEU2 CAN1* | This work | 6F |
| wM4rev1CAN-11D | *MATa MCM4VC::HIS3 rev1∆::Nat bar1∆::LEU2 CAN1* | This work | 6F |
| wrad52∆ | *MATa rad52∆::Hyg* | [1] | S4A |
| wtb-2A | *Mat a bar1∆::LEU2* | This work | S4A, S4E, S5A, S5D |
| wM4vc-4C | *MATa MCM4VC::HIS3 bar1∆::LEU2* | This work | S4A, S5B |
| wR4vn-1D | *MATa RNR4VN::Kan bar1∆::LEU2* | This work | S3A, S3B, S3C, S4A, S4C, S4D, S4E, S5A, S5B |
| wR4vnM4vc-11B | *MATa RNR4VN::Kan MCM4VC::HIS3 bar1∆::LEU2* | This work | S4D, S5A |
| W303sml1-10B | *MATa sml1∆::URA3* | [1] | S4C |
| wR4vnsml1-20B | *MATa RNR4VN::Kan sml1∆::URA3 bar1∆::LEU2* | This work | S4C |
| wR4VNM4VC-3D | *MATa RNR4VN::Kan MCM4VC::HIS3 bar1∆::LEU2* | This work | S5B |
| wrad51∆-2 | *MATa rad51∆::KanR bar1∆* | This work | S1 |
| wcaf4-2 | *MATa caf4∆::KanR* | This work | S1 |
| wcaf40-1 | *MATa caf40∆::KanR* | This work | S1 |
| wcaf130-1 | *MATa caf130∆::KanR* | This work | S1 |
| wccr4-3 | *MATa ccr4∆::KanR* | This work | S1 |
| wcrt10-2 | *MATa crt10∆::KanR* | This work | S1 |
| wdun1-1 | *MATa dun1∆::KanR* | This work | S1 |
| wrnr3-2 | *MATa rnr3∆::KanR* | This work | S1 |
| wrnr4-2 | *MATa rnr4∆::KanR* | This work | S1 |
| w303p-16A | *MATa +p414GAL1RNR1 (trp)* | This work | S2B |
| wM4p-15B | *MATa MCM4VC::HIS3 +p414GAL1RNR1 (Trp)* | This work | S2B |
| wRnr4GFP-1C | *MATa RNR4GFP::URA3 pep4∆::ADE2* | This work | S3C |
| wM4VCSCE-8B | *MATa trp1::(his3∆3'-his3∆5'::URA3) MCM4VC::Hyg bar1∆::LEU2* | This work | S8 |
| wM4VCSCErev1-1C | *MATa trp1::(his3∆3'-his3∆5'::URA3) MCM4VC::Hyg rev1∆::Nat bar1∆::LEU2* | This work | S8 |

1. González-Prieto R, Muñoz-Cabello AM, Cabello-Lobato MJ, Prado F. Rad51 replication fork recruitment is required for DNA damage tolerance. EMBO J. 2013;32: 1307–1321. doi:10.1038/emboj.2013.73

2. Cabello-Lobato MJ, González-Garrido C, Cano-Linares MI, Wong RP, Yáñez-Vílchez A, Morillo-Huesca M, et al. Physical interactions between MCM and Rad51 facilitate replication fork lesion bypass and ssDNA gap filling by non-recombinogenic functions. Cell Rep. 2021;36: 109440. doi:10.1016/j.celrep.2021.109440
