# Supplementary material for: Physical interactions between specifically regulated subpopulations of the MCM and RNR complexes prevent genetic instability
Source: PLoS Genet. 2024 May 22;20(5):e1011148. doi: 10.1371/journal.pgen.1011148 (PMC11149843; doi:10.1371/journal.pgen.1011148)
Supplement: S2 Table — DNA sequences and Figures panels where they have been used are indicated. (DOCX) [file pgen.1011148.s011.docx]

**Table S2. Oligonucleotides used in this study.**

| **Oligo** | **Sequence** | **Figure** |
| --- | --- | --- |
| MATa Distal 1 | CATGCGGTTCACATGACTTTTGAC | *MAT* probe |
| MATa Distal 2 | AGGATGCCCTTGTTTTGTTTACTG | *MAT* probe |
| Act1 probe 1 | CGAACAAGAAATGCAAACCGC | *ACT1* probe |
| Act probe 2 | CTTGTGGTGAACGATAGATGG | *ACT1* probe |
| For RNR1 | CCGGTACTCCAAAACCTCAA | *RNR1* qPCR |
| Rev RNR1 | CCACCAGCAGTTTTGGAAAT | *RNR1* qPCR |
| For RNR4 | GAAGCCGTGGAAATTGAAAA | *RNR4* qPCR |
| Rev RNR4 | GACCGTCAGCGACAAATTCT | *RNR4* qPCR |
| ACT1 F | TCGTTCCAATTTACGCTGGTT | *ACT1* qPCR |
| ACT1 R | CGGCCAAATCGATTCTCAA | *ACT1* qPCR |
| Up ClaI Rnr4 | TCGGACATCGATGTTCTAGCACACTGAAAG | p314RNR4 cloning |
| Lo BamHI Rnr4 | AGGTCAGGATCCTGCTATGTACCTCTATCT | p314RNR4 cloning |
| Up Mcm4MN | CGAGGGTGTAAGGAGATCAGTTCGCCTGAATAACCGTGTCCGGATCCCCGGGTTAATTAA | Mcm4-VC tagging |
| Lo Mcm4MN | TTATTAATTGTTACGCAGGGAATGATTGTAGTAGACAGCAGAATTCGAGCTCGTTTAAAC | Mcm4-VC tagging |
| Up Dun1 tagHA | CAATAAAATACCCAAAACATACTCAGAATTATCTTGCCTCCGGATCCCCGGGTTAATTAA | Dun1-HA tagging |
| Lo Dun1 tagHA | CCAGATTCAAACAATGTTTTTGAAATAATGCTTCTCATGTGAATTCGAGCTCGTTTAAAC | Dun1-HA tagging |
| Up Ccr4 tagHA | ATTTGAATTTATGAAGACAAACACAGGCAGTAAGAAAGTACGGATCCCCGGGTTAATTAA | Ccr4-HA tagging |
| Lo Ccr4 tagHA | GTACAGAGAGGAGGGAGGGAGTGGGATGAAAGTGTGCGGTGAATTCGAGCTCGTTTAAAC | Ccr4-HA tagging |
| Up Rnr4 tag HA | CGCTACCCCATCCAAGGAAATTAACTTTGATGATGACTTCCGGATCCCCGGGTTAATTAA | Rnr4-VN tagging |
| Lo Rnr4 tag HA | AAAAGTGGCCAAGAATAAAAGAACGCACCCCGTCGTTGACGAATTCGAGCTCGTTTAAAC | Rnr4-VN tagging |
| Up GFP-NLS | TGCTGCTGGTATTACCCATGGTATGGATGAATTGTACAAACGCTCAGGCCCCCCTAAGAA | Mcm4::GFP-NLS tagging |
| Up GFP-svnls3A2 | TGCTGCTGGTATTACCCATGGTATGGATGAATTGTACAAACGCAGCGGCCCACCAGCTAA | Mcm4::GFP- svnls3A2 tagging |
| Lo Mcm4-Kan | TTATTAATTGTTACGCAGGGAATGATTGTAGTAGACAGCAGAATTCGAGCTCGTTTAAAC | Mcm4::GFP-NLS and Mcm4::GFP- svnls3A2 tagging |
| Up Rnr4 F5 GFP | CGCTACCCCATCCAAGGAAATTAACTTTGATGATGACTTCGGTGACGGTGCTGGTTTA | Rnr4-GFP tagging |
| Lo Rnr4 R3n GFP | AAAAGTGGCCAAGAATAAAAGAACGCACCCCGTCGTTGACAGCGACCAGCATTCACAT | Rnr4-GFP tagging |
| NLSsv40 | ATAGCTCGAGTGCATATGCGCTCAGGCCCCCCTAAGAAAAAAAGAAAAGTAGAGGGAGGCTCTGGTCCACCTAAAAAAAAGAGGAAGGTCGAAGGGGGAAGTCGTTAGGGATCCAAGTCCTGCAGC | gBlocks |
| svnls3A2 | ATAGCTCGAGTGCATATGCGCAGCGGCCCACCAGCTAAAGCGGCCAAAGTTGAAGGTGGCTCTGGGCCACCCGCAAAAGCCGCGAAGGTTGAAGGTGGGTCCCGTTAGGGATCCAAGTCCTGCAG | gBlocks |
