# Supplementary material for: Physical interactions between specifically regulated subpopulations of the MCM and RNR complexes prevent genetic instability
Source: PLoS Genet. 2024 May 22;20(5):e1011148. doi: 10.1371/journal.pgen.1011148 (PMC11149843; doi:10.1371/journal.pgen.1011148)
Supplement: Attachment — Submitted filename: renamed_4a54d.docx [file pgen.1011148.s013.docx]

First, we want to thank the reviewers for their contribution to the improvement of the manuscript. We appreciate their effort and positive criticisms that we have tried to include either by additional experiments or modifications in the main text.

**Reviewer #1:** Review Summary:

Using proteomics, microscopy, genetics and various molecular biology approaches, the authors identified a novel physical interaction between the MCM helicase and ribonucleotide reductase RNR, and characterized the importance of this interaction for preventing genome instability. This manuscript is centered around the themes of spatio-temporal control of complexes and DNA synthesis, and the physical interaction between MCM and RNR involves small subpopulations. These interactions are independent of subcellular localization of the major pools of MCM and RNR in a cell and increase in response to exogenous DNA damage. Partial disruption of this interaction impairs the release of Rad52 from DNA repair centers, but RPA release does not occur. In the absence of the MCM-RNR interaction, Rad52 persists at repair centers and causes genome instability in the form of hypermutation and hyperrecombination.

As MCM was previously shown to interact with Rad51 and Rad52, the authors looked for other MCM interactors in the absence or presence of MMS. These results demonstrate novel roles for MCM and RNR, independent of their well-characterized roles in DNA replication and dNTP synthesis. The authors used the Mcm4-VC construct, which was partially defective in interacting with all RNR components, as a tool to study partial disruption of the MCM-RNR complex. This allowed them to study the biological consequences of loss of the MCM-RNR complex.
The results are original and of importance to researchers in the fields of DNA replication and repair, and these findings will be of broader interest to researchers in the field of genome integrity. The high conservation of the MCM and RNR complexes supports the possibility that that the results presented here using budding yeast as a model system should be of relevance to those studying mammalian systems.

The methodology is rigorous and the authors provide excellent detailed descriptions of the experiments performed and the rationale for each one. The authors also provide substantial evidence for their conclusions.

We appreciate the constructive comments of the reviewer and his/her suggestions to improve the manuscript.

The manuscript could be strengthened as follows:

There are many concepts described in detail in the Results section that are not introduced in the Introduction. In some sections, this makes it difficult to relate the results back to the original goals of the work and the bigger picture. In my opinion, moving some of the detailed background currently found in the Results to the Introduction section would streamline the manuscript.

We agree that the Introduction is focused in introducing MCM and RNR complexes and regulation, whereas the information about the DNA repair processes and centers was described in Results, interrupting the linearity of the arguments. We have followed the reviewer’ advice and moved those aspects to Introduction.

It would be helpful to have a model (could be panel in Figure 6) that gives a summary of the physical interactions, cellular localization (with respect to cell cycle stage and DNA damage), and biological roles for MCM, RNR and the MCM/RNR complexes. In some of the sections in the text, the descriptions became a very detailed and it becomes difficult to tie concepts together. A model or graphical abstract that summarizes the localization, subcomplexes, functions of the major complexes as well as those identified here would help to orient readers, especially non-experts.

Done as suggested. A graphical abstract summarizing the major finding with a descriptive legend has been included in Figure 6G.

There are two typos where Rnr3 has been incorrectly written as Rrn3 (page 5 second paragraph and page 18 second paragraph).

Corrected

The authors measured spontaneous mutagenesis by determining the CAN1R frequency in their various strains in Figures 6E and F. Were any of these CANR mutants sequenced to determine the what mutations are formed following failure of the MCM-RNR interaction? This mutation specificity could provide evidence as to the source of these mutations (TLS, Polymerase delta, etc) and/or the output of repair processes due to excessive Rad52 at DNA repair centers and inefficient PCNA loading (as suggested on page 19 of the Discussion).

We did not sequence canavanine-resistance mutants because we checked the dependence of the mutagenesis process on Rev1 in Figure 6F. The result demonstrated that all mutations arising in the *MCM4-VC* mutant are produced by polymerase ζ, suggesting that they result by TLS. To clarify this, now it reads: “The *MCM4-VC* was proficient in TLS; actually, it displayed a strong increase in spontaneous (10-fold), MMS (6-fold) and HU-induced (5-fold) Rev1-dependent mutagenesis (Figures 6E-F), suggesting that replicative DNA lesions are bypassed by Pol ζ-mediated TLS”.

The authors should discuss the possible other biological consequences of persistent Rad52 at sites of DNA repair. For example, could this prevent the binding of other proteins? These would be ideas to be explored in future work.

We think that the most likely consequence of Rad52 persistence is the increase in genetic instability (hypermutagenesis and hyperrecombination) as we argue in Discussion. How this occurs is unknown, but it might be linked to altering (more than preventing because in that case we would expect a defect in DNA repair or checkpoint activation) the correct binding of proteins to DNA. Additionally, Rad52 persistence might affect the function of Rad52 at other compartments, like its interactions with MCM at the nucleoprotein scaffold. These ideas are now Discussed as suggested. Now it reads: “Altogether, these results suggest that excessive Rad52 at the DNA repair centers alters the output of the repair processes leading to genetic instability, may be by altering the correct binding of DNA repair proteins at DNA lesions. Alternatively, Rad52 accumulation at DNA repair centers might affect its function at other compartments, like its interactions with MCM at the nucleoprotein scaffold”

**Reviewer #2:** This manuscript describes a novel set of interactions between the MCM complex and components/regulators of RNR. Temporal and spatial aspects of one of these interactions is analyzed using Mcm4 and Rnr4 fused to chimeric Venus fluorescent tags. A peculiar situation is observed where expression of the Mcm4-VN fusion protein appears to perturb the RNR interaction. The Rnr4-VN fusion, however, potentiates the interaction. At this point, the authors transition to using Mcm4-VN as a reagent to probe the significance of the RNR interaction, employing the logic that if a Mcm4-VN phenotype is ameliorated by Rnr4-VN it can be attributed to an interaction defect. Such discrimination becomes important, as Mcm4-VN exhibits phenotypes likely arising from perturbing MCM function in DNA replication.

The two primary phenotypes observed with Mcm4-VN that can be rescued (to variable extents) by Rnr4-VC are a delay in the disappearance of Rad52 foci following DNA damage/replication stress and an elevation in mutations attributable to TLS. There is also an ~ 2X increase in dNTP pools that is mildly suppressed by Rnr-VC.

All told, there is a lot of work here with a robust set of data. The first part of the manuscript characterizing the interactions could easily be reworked to stand on its own, albeit without the functional connection. The second part of the paper seems ambitious, painting some interesting yet broad strokes that go beyond the data shown. A main concern, of course, is interpretation of the Mcm4-VN mutant. In addition, the central phenotypes do not necessarily coalesce into a clear functional model.

We appreciate the constructive review and suggestions to improve the manuscript. We are aware about the limitations of the functional part that do not allow to infer or figure out how the MCM/RNR interactions regulate (directly or indirectly) the processes of Rad52 dynamics and mutagenesis. The work is centered in the description and characterization of the MCM/RNR interactions in response to DNA damage and their connection with two processes associated with genome integrity. We discuss several possibilities that might explain how an unscheduled accumulation of Rad52 at the repair centers might increase mutagenesis and recombination, but we agree that these two processes are not necessarily connected to each other, a point that we have clarified in Discussion. Now it reads: “Altogether, these results suggest that excessive Rad52 at the DNA repair centers alters the output of the repair processes leading to genetic instability, may be by altering the correct binding of DNA repair proteins at DNA lesions. Alternatively, Rad52 accumulation at DNA repair centers might affect its function at other compartments, like its interactions with MCM at the nucleoprotein scaffold. However, we cannot exclude the possibility that the defect in Rad52 release from the repair centers and the increase in genetic instability were independent of each other”.

Concerning the interpretation of the Mcm4-VC data, it stems from three genetic arguments:

1. A mutant defective in the MCM/RNR interaction (*MCM-VC*) causes a phenotype (Rad52 release; hypermutagenesis)

2. Both the interaction defect and the phenotype can be suppressed by a second mutation (*RNR4-VN*)

3. This suppression is not due to squelching of Mcm4-VC (and elimination of a putative dominant negative effect). This is an important point we missed in our previous version that we have included now as suggested by the reviewer (see below)

We think that they provide a strong genetic argument to propose that the loss of MCM/RNR interactions causes those phenotypes, as stressed now in the text. We do not know how or why, even though we speculate with potential possibilities for future studies. But the relevance of the study is restricted to the description of an unknown set of DNA damage-regulated physical interactions between MCM/RNR that somehow influence genome integrity, which we think is relevant for the field. We have tried to eliminate any overstatement in this regard.

Specific comments on first part of paper:

1) One question re first part of the manuscript is whether the shifted form of Rnr4 is tied into the observation that, as I followed, Dun1 is required for the interaction with the R2 subunits.

We do not think so because most Rnr4-VN is modified, not only the one that interacts with Mcm4 (e.g., see input in Figure 4). Actually, as it now better clarified in the characterization of the Mcm4-VC/Rnr4-VN, the massive recruitment of Mcm4 by Rnr4-VN is unlikely to be due to this putative modification; the unmodified Rnr4-VN also interacts with Mcm4-VC but the signal is much lower because most of Rnr4-VN is modified (Now it reads: “second, this potentially post-translationally modified Rnr4-VN* chimera has a MMS-independent strong affinity for Mcm4-VC (Figure 3B, IP panels). However, unmodified Rnr4-VN also interacted with Mcm4-VC in a MMS-independent manner despite was hardly detected in the input (Figure 3B, over-exposed Rnr4/Rnr-VN signals in the IP panels), suggesting that the strong interaction with Mcm4 is associated with the Rnr4-VN chimera that for unknown reasons becomes modified”.

Finally, this modification is observed only for Rnr4-VN and not for Rnr4. Accordingly, we have observed that the Rnr4-VN* modification is independent of Dun1 (see below). However, we think that this result is not necessary by the exposed reasons and could be confusing so we would prefer not to include it.

2) As I followed, the only evidence that RNR binds to the MCM hexamer and not just Mcm4 is the supplemental figure with Mcm7. Is that correct?

That is correct; we think that the fact that the RNR components interact with two different subunits of the MCM complex suggests that the interaction encompasses the whole complex. However, we cannot exclude that the interactions take place with individual subunits of the complex or with a non-canonical form of this, as we cannot exclude either that the MCM subunits interact with non-canonical forms of RNR (actually, Rnr2 interacts strongly with MCM in the absence of Rnr4). We have now clarified this point in the text. Now it reads in Discussion: “We cannot, though, discard the possibility that the interactions take place between some subcomplexes or non-canonical forms of the MCM and RNR complexes”.

3) I thought “these results indicate that the Mcm4- VC/Rnr4-VN complex is mobilized from the nucleus to the cytoplasm independently of the individual MCM and R2 complexes” was an overstatement. The data suggest the timing is different, but, for example, this could reflect a differential susceptibility of the fraction to the same form of regulation.

We agree with this interpretation and have modified the text accordingly. Now it reads “Therefore, the Mcm4-VC/Rnr4-VN complex and the individual MCM and R2 complexes display different kinetics of mobilization from the nucleus to the cytoplasm, which may reflect different regulation or susceptibility to the same regulatory mechanism”.

Specific comments on second part of paper:

1) Are Mcm4-VN phenotypes recessive? It seems possible a pool of Mcm4-VC interferes with a function that is separable from the interaction with RNR, but the enhanced interaction provided by Rnr4-VN titrates Mcm4-VC away from this pool. In such a situation, Mcm4-VN might act in a dominant negative manner.

This is an important possibility we had not explored. To analyze it, we have studied the three major phenotypes of Mcm4-VC (MMS and HU sensitivity, Rad52 foci accumulation and hypermutagenesis) in cells (wild type and *MCM-4-VC*) transformed with a centromeric plasmid expressing either a wild type copy of Mcm4 from its own promoter or an empty vector as control. The results clearly indicate that the *MCM4-VC* allele is recessive for the mentioned phenotypes (Figures S4A, S5E and S6B). The need for this control has been included in this new version when explaining the requirements a phenotype has to fulfill to be associated with a loss of MCM/RNR interactions in the *MCM4-VC* mutant. Now it reads: “This recovery experiment is essential to genetically associate a phenotype with the loss of MCM/RNR interactions, as the tagging of the Mcm4 with VC can alter some other functions hampering the interpretation of the results. In addition, it is important to demonstrate that the *MCM4-VC* allele is recessive for the analyzed phenotype to rule out the possibility that this phenotype was due to a dominant negative effect of Mcm4-VC separable of its interaction with RNR and titrated away by Rnr4-VN in the double mutant *MCM4-VC RNR4-VN*”.

Is Rnr3 constitutively upregulated in the Mcm4-VN mutant?

It is (new Figure S7B), as expected by the accumulation of spontaneous DNA damage in *MCM4-VC* cells that is independent of the loss of interaction with RNR. We indicate in the last section of Results that the increase in dNTPs in this mutant in the absence of genotoxic agents (Figure S7A) was likely influenced by the accumulation of spontaneous DNA damage. This is consistent with the reduction in the frequency of petite formation (Figure S4D) and the accumulation of Rnr3 reported for this new version (new Figure S7B). Now it reads. “These levels augmented approximately two times in the *MCM4-VC* mutant in the absence of genotoxic agents (Figure S7A). This increase is likely influenced by the accumulation of spontaneous DNA damage in this mutant as supported by the reduction in the frequency of petite formation and the accumulation of Rnr3 (Figures S4D and S7B)”.

2) Given the phenotypes associated with Mcm4-VN, the authors should comment on whether this introduces caveats in using Mcm4-VN as a physiological reporter for the Rnr4 interaction.

We agree that the genetic instability phenotypes that are not associated with the loss of interaction with RNR have to be taking into account for a right interpretation of the results, and that future studies will benefit from using cleaner mutants. Thus far, though, it is not easy to define a strategy to search for mutants that specifically disrupt the MCM/RNR interaction, reason by which we have used the *MCM4-VC* mutant.

It may be stressed that despite having these obvious limitations, the fact that we can partially rescue (even artefactually) the interactions in the *MCM4-VC/RNR4-VN* double mutant provides a unique genetic opportunity to confirm that the phenotypes are associated with the loss of the MCM/RNR interaction.

We have indicated the limitations and advantages of the Mcm4-VN construct when explaining the logic behind using this chimera to study the relevance of the MCM/RNR interactions. Now it reads: “These results open the possibility of using the *MCM4-VC* strain to study the effect of partially disrupting the MCM/R2 interactions. Importantly, to confirm that a defect induced by Mcm4-VC is due to a loss of interactions with RNR components and not to another MCM function, this defect should be at least partially recovered in cells expressing Mcm4-VC and Rnr4-VN where the MCM/R2 interactions are “artefactually” restored. This recovery experiment is essential to genetically associate a phenotype with the loss of MCM/RNR interactions, as the tagging of the Mcm4 with VC can alter some other functions hampering the interpretation of the results.”

3) The blots shown in Figure 3B need a more comprehensive description in the text or legend. Different antibodies are used to detect the Mcm4 and Rnr4 fusions. Nonetheless, the apparent massive increase in recovery of Rnr4 relative to Mcm4 IP levels is notable. Some regions of the bands, zoomed in, appear pixelated whereas other areas do not. Is this a saturation effect? The bottom RNR panel, and how it relates to the highly shifted form, needs a clearer explanation.

The text has been explained with more detail to make clearer the different proteins and their electrophoretic properties. Likewise, we have clarified that the bottom RNR panel is an over-exposition of part of the upper panel to show that the unmodified Rnr4-VN chimera can also interact with Mcm4-VC. Indeed, the fact that unmodified Rnr4-VN also interacted with Mcm4-VC in a MMS-independent manner despite was hardly detected in the input (Figure 3B, over-exposed Rnr4/Rnr-VN signals in the IP panels) suggests that this strong interaction is not associated with the Rnr4-VN* modification. We do not know why the Rnr4-VN chimera displays such a strong interaction with Mcm4-VC; a stabilization of the interaction through the VC and VN domains is likely involved but it is not sufficient to explain the massive recruitment by Mcm4-VC because Rnr4-VN also strongly interacts with wild type Rnr4 (Figures 4). These arguments have been included in the description of Figures 3B and 4. In any case, as mentioned above and in the arguments to use Mcm4-VC as a tool to study the relevance of the MCM/RNR interactions, the possibility of using this “artefactually” generated MCM/RNR complex allowed us to perform recovery experiments to genetically associate the observed phenotypes with the loss of MCM/RNR interaction.

I guess the reviewer refers to the Mcm4/Mcm4-VC IP signal (+MMS) (top right panel). By mistake, we selected a saturated image. We have replaced it with a lower exposition.

4) The discussion emphasizes a primary role for the MCM/RNR interaction is likely to be (apparently negative) regulation of dNTP levels. The main result is that the Mcm4-VN fusion has persistent Rad52 foci. How those two things are interconnected is at present unclear, giving that part of the analysis a preliminary feel.

We guess this has not been interpreted correctly. We do not claim that a primary role for the MCM/RNR interactions is to regulate the pool of dNTPs, and more importantly, we have not connected dNTPs with the Rad52 foci or mutagenesis phenotypes. Actually, we insist in that these phenotypes are not associated with alterations in dNTPs. In the last section of Results, when studying the possibility that the phenotypes were associated with an unbalance in dNTPs, we claim: “These levels (dNTPs) augmented approximately two times in the *MCM4-VC* mutant in the absence of genotoxic agents (Figure S7A). This increase is likely influenced by the accumulation of spontaneous DNA damage in this mutant as supported by the reduction in the frequency of petite formation and the accumulation of Rnr3 (Figures S4D and S7B); however, the pool of dNTPs was slightly but significantly lower in the double *MCM4-VC RNR4-VN* than in the single *MCM4-VC* mutant, pointing to a putative role for the MCM/R2 interactions negatively regulating the levels of dNTPs. In contrast to the situation in the absence of genotoxic agents, the partial loss of MCM/R2 interactions in the *MCM4-VC* mutant did not affect the concentration of dNTPs in the presence of 0.05% MMS (Figure S7), discarding the possibility that Rad52 foci persistence and genetic instability in this mutant were associated with unbalanced dNTPs levels.” We stress this point in the Abstract, Results and Discussion. Actually, we discuss some examples in the literature in which RNR controls cellular processes independently of dNTPs. What we intended to mention at the beginning of the Discussion was that a simple explanation for the fact that MCM interacts with RNR and several of its regulators would be to regulate dNTPs. However, in accordance with our results, we state in Discussion that “A simple explanation for the functionality of these interactions would be that they modulated the RNR activity in response to DNA damage, and actually the *MCM4-VC* mutant displays a slight increase in the pool of dNTPs that seems to be associated with a partial loss of the MCM/R2 interactions. However, this increase was not observed in response to 0.05% MMS, which causes more DNA damage and checkpoint activation than the expression of the Mcm4-VC protein. Therefore, at this point, we cannot conclude that the MCM/RNR interactions have a role in controlling the levels of dNTPs”. We have introduced some additional modifications in the text to make this important point clearer.

5) Similarly, I also did not follow how the previous work on a non-HR role for Rad52 in TLS specifically informs a possible connection between dNTP levels and dispersal of Rad52 from foci. Rightly or wrongly, I was left feeling that a synthesis of ideas is envisioned that is not yet supported by the current data, evocative as it may be.

We want to highlight that we do not connect hypermutagenesis with dNTP levels at any moment. In results, we first document a defect in the release of Rad52 from DNA repair centers. Then, we discard that this defect was due to impaired homologous recombination. Then, because Rad52 plays a non-recombinogenic role in TLS that can be visualized by an accumulation of Rad52 foci in TLS mutants, we analyze TLS and observe no defect (actually an increase in mutagenesis). Finally, we ask if these phenotypes might be associated with alterations in dNTPs, and we conclude that they are not. In Discussion, we claim that “These results suggest that the MCM/R2 interactions might be required to maintain the attractive potential that retains Rad52 at the droplet. At this point we do not have any clue about how this is achieved, except that is not mediated by alterations in the pool of dNTPs”. We have reviewed the text to eliminate any possible ambiguity about this.

6) A model figure with what is envisioned by “hub” would contribute to the paper. What would be the advantage of compartmentalizing/complexing the RNR and signaling factors together in this fashion?

As Discussed, we do not know how or why these MCM/RNR interactions control Rad52 persistence at repair centers and genetic stability. We speculate about different possibilities, like signaling factors to control the Rad52 attractive potential or more indirect scenarios as those reported for MCM and RNR regulating the availability of mRNAs and/or proteins by maintaining membrane-less organelles. We do not dare to speculate more about what advantages these interactions provide without more data. The major contributions of this study are the description of the MCM/RNR interactions and their genetic connection with Rad52 dynamics and genome integrity (it is also important of mention that we describe for the first time to our knowledge that Rad52 foci resolution is not necessarily an indication of DNA repair).

As also suggested by reviewer 1, a model figure summarizing the major findings has been included in Figure 6G.

**Reviewer #3:** The study by Yáñez-Vilches and coworkers investigates the interaction between Mcm4 and several subunits of the ribonucleotide reductase complex (Rnr). A proteomic interaction screen with Mcm4-GFP in the presence and absence of low-dose MMS suggests that Dun1 and Rad53 show increased binding with Mcm4, and regulators of Rnr. The volcano plots that are shown would suggest that Rnr3 binding is not significantly enhanced after MMS treatment. The way this is described in the manuscript is ambiguous (page 5). Nevertheless, the authors show by pulldown of Mcm4-GFP that Rnr can be co-immunoprecipitated. The claim that the interaction is enhanced by MMS is only visible on the blot in Figure 1E; the other blots suggest that there is little or no change.

Concerning this first point, we start by mentioning the most relevant proteins that appeared in the proteomic screening, which include factors that are associated with RNR regulation like Rad53, Dun1 and Rnr3 (that was detected with low statistical significance, although with a high fold increase relative to the untagged control). The next step in the argument is to confirm the interactions by CoIP. Once validated, the whole data were a solid base to explore if the interactions also included the rest of components of the RNR complex, which might be lost in the proteomic analysis by technical reasons. We have revised the text to get rid of any ambiguity about why we decided to study the MCM/RNR interactions.

The increase in the interactions between MCM with Dun1 and RNR components is clear in all blots: Dun1 (Figure 1C), Rnr1 and Rnr3 (Figures 1D, 1F and S2A, left) and Rnr2 and Rnr4 (Figures 1E, 2F, 3B and S2A, right), even though, as mentioned in the text, “the DNA damage-induced expression might explain totally or partially the increase in the interaction with MCM of Rnr1 and Rnr4, respectively, but not of Rnr2” (see blots in Figure 1E, 2F, 3B and S2A, right). These figures are the only ones in which a comparison between +/- damage can be done; in Figures 1F, 1G, 1H, 2D and 2E, +/- DNA damage was not performed in parallel (it was not necessary because the comparison had already been done), and the expositions are not comparable. May be the confusion stems from here. We have clarified this for Figure 2D, where +/-DNA damage are shown. In the rest of cases, each analysis is shown in a different Panel Figure (experiment) and is not comparable. Likewise, it has been indicated in the legend for Figure 3B that the IP samples were run into different gels due to space limitations but treated in parallel, and therefore the signals are comparable. We hope the effect of DNA damage on the interactions is clearer now.

The authors show numerous experiments that alter the cellular localization of Mcm4 and do not find that this affects the localization of Rnr. They conclude that only a small subpopulation of Mcm4 and Rnr subunits bind to each other. They subsequently tag Mcm4 and Rnr4 with the N- and C-terminal parts of the Venus fluorophore, respectively. With this sensitive interaction measure they confirm that small fractions of Mcm4-VC and Rnr4-VN interact in the nucleus in G1 and then gradually accumulate in the cytoplasm in S/G2. Although this is interesting, it remains unclear what the biological significance of this result is. The localization does not significantly change under DNA damage conditions.

Even though it is somehow expected taking into account the known functions of the MCM and RNR complexes, we think it is important to demonstrate that the interactions affect to a small subpopulation of both complexes, which may explain the specific functions of these interactions. We also agree that the biological significance of the cellular location of the interactions remains unknown and will require future studies for a better understanding of the process, and that the lack of effect in response to DNA damage might be an artefactual effect of the employed chimeras, as discusses in the text. Still, we think that this description is interesting and meaningful in this biochemical characterization that for the first time reveals DNA damage and Dun1-regulated physical interactions between the MCM and RNR complexes, which is the major finding of the first part of the paper. We think that these biochemical findings, by themselves, open a new and worth to explore field.

Furthermore, the authors establish that Mcm4-VC is compromised in binding untagged Rnr4. Mcm4-VC expression causes low level spontaneous damage that cannot be suppressed by Rnr4-VN expression. The authors then investigated whether the Mcm4 mutation affects double strand break (DSB) repair kinetics. However, Mcm4-VC expressing cells appear to repair DSBs with wildtype kinetics. Only when cells are the authors studied both RAD52 and RPA foci did they see a slight delay in the release of Rad52 from DNA after DSB repair. Release of Rad52 was similar in Mcm4-VC and Mcm4-VC Rnr4-VN strains arguing that the interaction between Mcm4 and Rnr subunits does not have a significant role in repair kinetics.

As the referee mentions, the kinetics of DSB repair is wild type for all strains, indicating that the interaction between Mcm4 and Rnr subunits does not have a significant role in repair kinetics. Also, the release of Rad52 from the foci was impaired in Mcm4-VC expressing cells. However, Rad52 release was not similar in Mcm4-VC and Mcm4-VC Rnr4-VN strains arguing that the interaction between Mcm4 and Rnr subunits regulates the release of Rad52 from the repair centers. It is not clear to us what is the criticism here.

Because it is unclear why Rad52 release is delayed in Mcm4-VC cells under the experimental conditions the authors use, I find it difficult to draw any strong conclusions from this part of the study. It might have nothing to do with the diminished binding to Rnr.

It is important to mention that the fact that we do not know how or why MCM/RNR interactions regulate those processes does not mean they do not do it. We provide genetic evidence that support a connection between the loss of the MCM/RNR interaction and the delay in the release of Rad52 (and also hypermutagenesis). First, it observed in Mcm4-VC cells that display a partial loss of MCM/RNR interactions; second, this phenotype is partially suppressed in Mcm4-VC/Rnr4-VN cells that partially restore the MCM/RNR interactions; and third (included in this new version of the manuscript in Figures S4A, S5E and S6B), the effect of Mcm4-VC is not dominant, discarding the possibility that the suppression effect of Rnr4-VC were not due to the restoration of the interaction with MCM, but because it were squelching Mcm4-VC and thus preventing a putative negative dominant effect of this allele separable from its interaction with RNR. These three are the genetic requirements that a phenotype has to fulfill to be associated with a loss of MCM/RNR interaction in the *MCM4-VC* mutant. These arguments have now been clarified in the text to highlight the fact that a loss of MCM/RNR interactions causes genetic instability phenotypes.

Even though we discuss some hypothetical possibilities, why or how the MCM/RNR interaction controls Rad52 release and hypermutagenesis is currently unknown and further experiments will be required. But it is clear from our results with the *MCM4-VC* strains that Rad52 release from DNA repair centers can be uncoupled from the molecular repair of the DNA lesion. This suggests a regulatory mechanism controlling Rad52 dynamics, which is consistent with the reported liquid-phase nature described for Rad52 at the DNA repair centers. This has been made clearer in the Discussion. We think that the description for the first time of the MCM/RNR interactions and its genetic connection with genetic instability provide an important advance for the field even without mechanistically demonstrating how this function is achieved.

In summary, I don’t find the conclusions as they are presented in the abstract justified by the data. The data show that there is an interaction between Mcm4 (and Mcm7) and Rnr subunits but whether the interaction that might be indirect or indirect serves any biological function remains elusive.

We have clarified in the text that the MCM/RNR interactions might be mediated by other proteins, which does not affect the conclusions of the manuscript. We have tried to clarify the reviewer’ concerns about the effect of DNA damage on the MCM/RNR interactions and the connection of these interactions with Rad52 dynamic in the repair centers. Following reviewer 2 advice, we have also discarded a dominant negative effect of Mcm4-VC on the observed phenotypes. Altogether, we think that the analyses of the *MCM-VC* strains allow us to propose that the MCM/RNR interactions play a biological role in genetic stability.
